# Interrogating the regulatory epigenome of cellular senescence

**DOI:** 10.1007/s00018-025-05848-w

**Published:** 2025-08-31

**Authors:** Dimitris-Foivos Thanos, Orestis A. Ntintas, Emmanouil I. Athanasiadis, Angelos Papaspyropoulos, Russell Petty, Vassilis G. Gorgoulis

**Affiliations:** 1 Molecular Carcinogenesis Group, Department of Histology and Embryology, Medical School, National and Kapodistrian University of Athens, Athens, Greece; 2 Intelligencia, Inc., New York, NY USA; 3 Medical Image and Signal Processing Laboratory (MEDISP), Department of Biomedical Engineering, University of West Attica, Athens, Greece; 4 Biomedical Research Foundation, Academy of Athens, Athens, Greece; 5 Ninewells Hospital and Medical School, University of Dundee, Dundee, UK; 6 Faculty Institute for Cancer Sciences, Manchester Academic Health Sciences Centre, University of Manchester, Manchester, UK

**Keywords:** Chromatin, Epigenetics, Cellular senescence, Epigenetic modulators

## Abstract

Chromatin, the spatial organizer of genomic DNA, is hierarchically folded into higher-order structures to facilitate DNA compaction, enabling genome surveillance. Understanding the organization and function of the three-dimensional (3D) genome is critical to profile chromatin accessibility and functional interactions that govern gene regulation across multiple biological processes, including aging and one of its hallmarks, cellular senescence. Cellular senescence constitutes a defensive stress response to various intrinsic and extrinsic stimuli, preserving cellular and organismal homeostasis through a generally irreversible cell cycle arrest. In this review article we discuss epigenetic alterations occurring to DNA and chromatin that drive and fuel the onset of this complex phenomenon. As such, we describe major large-scale chromatin events, including the formation of higher-order chromatin structures and the 3D spatial alterations of the genome that occur during senescence. We also discuss global heterochromatin loss, deficiencies in nuclear lamins, the depletion of core histones and their modifications, as well as the epigenetic regulation of the senescence-associated secretory phenotype (SASP), all of which serve key roles in the epigenome of senescent cells. To clearly demonstrate the significance of epigenetic modifications, data from a computational meta-analysis are presented, aiming to further underpin key epigenetic mechanisms occurring in senescent cells. Last, we highlight promising epigenetic modulators implemented in therapeutic strategies for senescent cell detection and elimination, possibly leading to significant clinical advances against various age-related diseases as well as the delay and prevention of the aging onset.

## Introduction

**Fig. 1:**
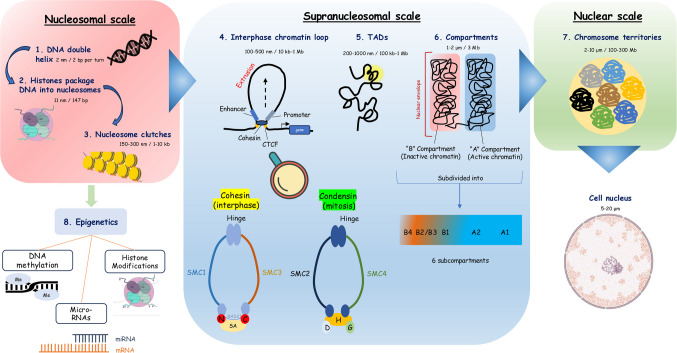
Overview of chromatin structure and organization. Chromatin folding follows a multifactorial, hierarchical pattern starting with the DNA double helix (1). The DNA is then wrapped around histones to form nucleosomes (2) which group together into nucleosome clutches (3). Chromatin fibers further fold into chromatin loops (4) held by cohesin during interphase or condensin during mitosis. These complexes are built upon heterodimerization of Smc2/4 (condensin) and Smc1/3 (cohesin). Self-interacting genomic regions called topologically associating domains (TADs) (5), characterized by the presence of CTCF, organize the genome into functional units. Long-range interactions between TADs contribute to chromatin compartmentalization resulting in the formation of A (active) and B (inactive) compartments (6), which are further subdivided into A1, A2 and B1, B2, B3, B4. Ultimately, chromosomes are compartmentalized into discrete territories (7). The functional complexity of DNA is further increased via epigenetic modifications (8) of the genome (DNA methylation, histone modifications and miRNAs) (See also text in Section “Chromatin organization”). Created with bioicons.com (modified histone icon by DBCLS https://togotv.dbcls.jp/en/pics.html is licensed under CC-BY 4.0 Unported https://creativecommons.org/licenses/by/4.0/) and nucleus icon by Servier https://smart.servier.com/ is licensed under CC-BY 3.0 Unported https://creativecommons.org/licenses/by/3.0/)

Chromatin is the structural assembly of DNA coiled around proteins, giving the genome its characteristic tightly-packed, non-linear form (Fig. 1) [1]. The level of condensation dictates the expression or suppression of genes, playing a critical role in several processes (DNA replication, repair, differentiation). Its folding follows a multifactorial, hierarchical pattern starting with the assembly of nucleosomes, followed by the organization into chromatin loops, topologically associating domains (TADs), chromosome compartments and, ultimately, chromosome territories [2]. The functional complexity of DNA is further increased via epigenetic modifications of the genome (Fig. 1) [3]. The chromatin status is an important aspect in cell senescence, a cellular state that has attracted significant attention in the last decades, due to the recognition of its complex roles in both aging and disease. Senescent cells undergo significant changes in their chromatin structure which impact genome accessibility and alter their transcriptional activity [4]. Epigenetic therapies targeting cell senescence might reduce the accumulation of senescent cells, presenting a novel strategy for treating age-related diseases [5]. In this review, we present a concise overview of chromatin organization, the methods used to study it, its role in dictating the senescence program, and potential therapeutic interventions to mitigate the adverse effects of senescence.

## Chromatin organization

### Nucleosomes and internucleosomal interactions

The main repeating building block of chromatin is the nucleosome (Fig. 1) [6]. The structure of the canonical nucleosome core includes an octamer of proteins, wrapped by a ~ 147 bp segment or 1.7 turns of DNA [6]. The protein components of the nucleosome are histones, and the octamers are made up of two copies each of histones H2A, H2B, H3, and H4, also known as core histones [7]. The short and linear piece of DNA between two neighboring nucleosomes is called linker DNA and it is protected by the linker histone H1 [8]. Flexible regions extending from the surface and flanking both ends of the histone fold are termed “histone tails” [9]. Histone tails contribute to nucleosome stability and facilitate genomic transcription as they are able to bind transcription factors and enzymes [9]. Contrary to prior observations supporting the folding of a nucleosomal array into a 30 nm fiber, recent studies using super-resolution microscopy revealed that nucleosomes appear more flexible, grouped in discrete domains known as “nucleosome clutches” (Fig. 1) [10, 11]. Linker histones contribute to the stabilization of the nucleosome-nucleosome and nucleosome-DNA interactions [12].

### Chromatin loops

Regulatory elements (promoters, enhancers, and silencers) comprise binding sites for transcription factors which may be positioned upstream or downstream of the genes they control [13]. Chromatin looping is widely acknowledged and explains the capacity of the above-mentioned elements to regulate transcription at a distance (Fig. 1) [14]. Within this context, the formation of enhanceosome loops, referring to specific DNA arrangements within a nucleoprotein complex comprised of multiple transcription factors, increases the level of gene expression [15]. Recent advancements in the field of chromosome biology indicate the existence of different types of chromatin loops depending on their structure and function. Chromatin looping is a key structural feature of chromatin organization and below are discussed the three major types of chromatin loops: mitotic loops, interphase loops and transcription loops [16].

#### Mitotic loops

The model of rod-shaped structures refers to chromosomes which are condensed during mitosis in order to facilitate successful chromosome segregation [17]. In this process, Structural Maintenance of Chromosomes (SMC) proteins play a crucial role as they orchestrate the mitotic chromatin restructuring landscape [18, 19]. SMC complexes constitute a large family of ring-shaped ATPases with notable members including condensin, cohesin, and the Smc5/6 complex. Condensin and cohesin complexes are built upon heterodimerization of Smc2/4 and Smc1/3, respectively, which is facilitated by the central “hinge” domain of the complex (Fig. 1) [20, 21]. On the other hand, the ATPase head domains interact with the kleisin subunit of the complex, recruiting additional subunits to it. In mitosis, greater than 500-fold compaction is achieved due to loop extrusion by condensin, which entraps DNA molecules within its ring and extrudes the loop until it encounters another condensin complex that halts the loop’s progression [22]. Thus, condensins form a central axis (scaffold) with loops emanating from it [23]. The faithful execution of segregation requires the interplay between condensin and topoisomerase II, which enables both condensation and relaxation of DNA supercoiling [22].

#### Interphase loops

Both condensin and cohesin are capable of compacting DNA in the presence of nucleosomes. Despite their distinct structure and function, cohesin contains conserved chromosomal ATPases of the SMC protein machinery [24]. During interphase, chromatin is organized into loops and TADs, which will be addressed later in this review. In the case of interphase loops, they are cohesin-dependent, where cohesin binds randomly on chromatin and catalyzes genome folding through bidirectional loop extrusion (Fig. 1) [19]. Thus, cohesin facilitates enhancers to scan for their target promoters. Loop domains are enriched in binding sites for the CCCTC-binding factor (CTCF) [18]. CTCF and cohesin co-occupy tens of thousands of binding sites across the genome, promoting the formation of basic three-dimensional structural loops [25]. These chromatin loops will initially be small and gradually expand until they encounter occupied CTCF DNA-binding sites [16, 26]. Recent studies, leveraging advanced methodologies, indicate that the CTCF-cohesin interaction is a multi-step mechanism in which cohesin temporarily pauses next to CTCF and then stabilizes exclusively on the N-terminal side of CTCF [26].

#### Transcription loops

The formation of transcription loops was first identified in lampbrush and polytene chromosomes in the 1880s [16]. In lampbrush chromosomes, extended lateral loops from the chromomeres (regions of compacted chromatin) represent units for the coordinated and intense transcriptional activity of a gene or set of genes [27]. Polytene or giant chromosomes are present in a wide range of insects, plants, animals, and unicellular organisms, and form loops called puffs through transcriptional activation [28]. More recent studies in mouse cells indicate the formation of open-ended transcription loops in highly expressed genes and demonstrate that RNA polymerases glide along these loops and carry nascent RNA molecules, which undergo RNA maturation, a typical processing step of eukaryotic RNA. These microscopically resolvable transcription loops mimic both lampbrush loops and polytene puffs [16, 29].

### Topologically associating domains (TADs)

As a result of loop extrusion, an additional key feature of eukaryotic genome folding is the organization into sub-megabase scale domains, also known as TADs (Fig. 1) [30, 31]. TADs are considered self-interacting genomic regions acting as functional units, with a low degree of interaction with regions outside of the domain [32]. TAD features appear to be highly conserved across mammalian species, with a key characteristic being the presence of the insulator-binding protein, CTCF, along with the SMC cohesin complex at most TAD boundaries [33, 34]. Within TADs, genes and their regulatory elements are often in close spatial proximity, ensuring efficient transcriptional regulation [2, 35]. In addition, specific active transcription marks, such as H3K4me3 and H3K36me3, are strongly enriched at TAD boundaries [33]. Of note, evidence indicates that TADs are enriched for housekeeping genes and transcription start sites (TSS), with depletion of insulatory TAD boundaries leading to ectopic gene expression *in vitro* and *in vivo* [32, 36]. Latest studies also demonstrate the importance of TADs, revealing that deletions of TAD boundaries disrupt normal genome function and cause abnormal gene expression, developmental defects, and tumorigenesis [31, 37, 38].

### Chromatin compartmentalization

Advancements in techniques for studying chromatin organization have opened new horizons in our understanding of long-range interactions between TADs [2]. These interactions can occur between very distant areas on the linear genome, giving rise to nuclear compartments which separate active (euchromatin) and inactive (heterochromatin) chromatin (Fig. 1) [39]. In more depth, the human genome is partitioned into either “A” compartments, which are associated with open chromatin and subsequently actively transcribed genes and active histone marks, or “B” compartments, which in contrast, correlate with closed chromatin, inactive genes and repressive marks [40]. A and B nuclear compartments are further subdivided into six smaller contact domains known as subcompartments (two for the A compartment and four for the B compartment) which arise through the attraction and/or repulsion between individual TADs with analogous epigenetic marks (Fig. 1) [41, 42]. The “compartment switch” process in genomic regions can be influenced by various factors such as epigenetic state, gene expression and replication timing (RT) [43]. Although A/B compartments are less well understood, current knowledge of chromatin organization indicates that they are cell-type specific and less conserved compared to TADs [44].

### Chromosome territories

Chromosome territories refer to the distinct regions occupied by chromosomes within the nucleus (Fig. 1) [45]. This major feature of nuclear architecture is conserved across evolution, appears cell-type specific and is characterized by the arrangement of chromosomes in a radial pattern within the nucleus [46]. The organization of chromosome territories is not random; instead, it is associated with both gene density and chromosome size [47]. The gene-rich chromosomes tend to be located in the interior of the nucleus, while chromosomes with a lower gene content are more commonly found at its periphery [48, 49]. In addition, chromosome territories are spheroid dynamic structures enabling gene relocation from the periphery towards the interior or the opposite [50, 51]. High-order spatial interactions between neighboring chromatin territories have been described using high-throughput techniques. These structural interactions affect gene expression and are crucial for cell fate determination [52].

### Epigenetic mechanisms

Chromatin is not an inert structure. Instead, DNA can respond to external cues influencing various biological processes [53]. Histone modifications, along with DNA methylation and micro-RNAs (miRNAs) are the main means of the epi-information beyond the DNA sequence, collectively referred to as epigenetics, the study of molecular modifications on DNA that regulate gene expression, without altering the DNA sequence (Fig. 1) [3].

The N-terminal tails of the histones can undergo post-translational modifications, which are the core mechanism of epigenetic regulation [54]. Histone post-translational modifications (HPTMs) include histone methylation, histone acetylation and histone phosphorylation. The addition of methyl groups usually to lysine (K) residues on histones H3 and H4, catalyzed by histone methyltransferases (HMTs), leads to histone modifications such as H3K4, H3K9, H3K27, H3K36, H3K79 and H4K20 [54]. Among them, H3K4, H3K36 and H3K79 are usually considered active marks as they are enriched in coding regions. These marks, are commonly associated with the activation of transcription making chromatin more accessible to transcription factors [55, 56]. In contrast, H3K9, H3K27 and H4K20 are generally considered as repressive marks, as they are associated with gene repression, heterochromatin and the formation of broad domains at promoters of silenced genes [57–60]. However, the outcome of the modification depends on the methylation site and methylation level (number of added methyl groups). For instance, H4K20me exists in three distinct forms mono-, di-, and trimethylation. H4K20me1 facilitates increased chromatin accessibility promoting expression of housekeeping genes, while H4K20me3 drives the maintenance of DNA methylation, promoting gene silencing [61, 62]. Lysine residues of histones are also subjected to acetylation, catalyzed by histone acetyltransferases (HATs) [53]. The most studied modification is H3K27ac, which usually co-exists with H3K4me3 and is defined as an active enhancer mark [63]. More specifically, H3K27ac is correlated with transcriptionally active genes and is found at active promoters and enhancers or around TSS [64]. Beyond the fact that H3K27ac distinguishes active enhancers from poised ones, it also contributes to cell identity control [65]. Notably, biological processes such as cell fate determination and differentiation are regulated by epigenetic dependent events [66]. The regulatory regions of development-associated genes that are marked by both activating and repressive histone modifications (such as H3K4me3-activating or H3K27me3-repressing) are known as “bivalent” or “poised” promoters [67]. Poised chromatin is associated with pluripotency and was first identified in embryonic stem cells, where transition of a subset of poised genes to a fully active state (resolution) occurred in response to external signals [66]. Characteristically, poising takes place during gametogenesis, silencing genes that are later activated at the onset of embryogenesis, overall acting as an epigenetic control mechanism over somatic tissue patterning [66]. Lastly, the deposition of phosphate groups onto serine (S), threonine (T), or tyrosine (Y) residues on histones is known as histone phosphorylation. Although this type of modification is less well-understood, it plays a role in gene expression, cell cycle regulation and DNA damage response [68].

DNA methylation is a heritable epigenetic mark in the mammalian genome, involving the addition of a methyl group to the C-5 position of the cytosine ring of DNA on genomic 5′-C-phosphate-G-3′ (CpG) dinucleotides, by a family of DNA methyltransferases (DNMTs) [69]. This direct chemical modification of the DNA in promoter regions is covalent and reversible and favors gene silencing by recruiting repressive proteins or inhibiting the binding of transcription factors to DNA [70]. Transcriptional regulation of the genome is ensured by the coordinated action of DNMTs, which often exhibit distinct methylation patterns [71]. DNA methylation regulates various cellular and organismal processes such as, chromatin-based transcriptional regulation, chromosome stability, X-chromosome inactivation and embryonic development. Nevertheless, disruptions in DNA methylation can result in the silencing of tumor suppressor genes or oncogene activation, thereby contributing to genomic instability in numerous human diseases, including cancer [72–75]. Moreover, there is a strong association between hypomethylation of retrotransposons (LINE-1 and Alu elements) with genomic instability in non-small cell lung cancer [76]. Hypomethylation seems to render tumors more susceptible to senescence signals. If so, cancer cells that are more sensitive than normal ones to chemotherapy could rely on senescence as a means to survive.

Discovered about 30 years ago, miRNAs are endogenous small non-coding RNAs that regulate genomic translation in higher eukaryotes [77]. In greater detail, miRNAs act as epigenetic modulators either by binding to a target mRNA, leading to its degradation or suppression, or by repressing transposons through heterochromatin formation [77]. Even though they do not cause changes in the DNA nucleotide sequence, the chromatin structure is impacted by their function, as miRNAs regulate histone modifier molecules including DNMTs (miR-29 family) and histone deacetylases (HDACs, e.g., miR-140) [78, 79]. miRNAs hold a fundamental role in biological processes such as cellular proliferation, differentiation, DNA repair and aging, and hence they can be used as validated diagnostic and prognostic biomarkers [80].

## The evolution of chromatin analysis techniques

**Fig. 2:**
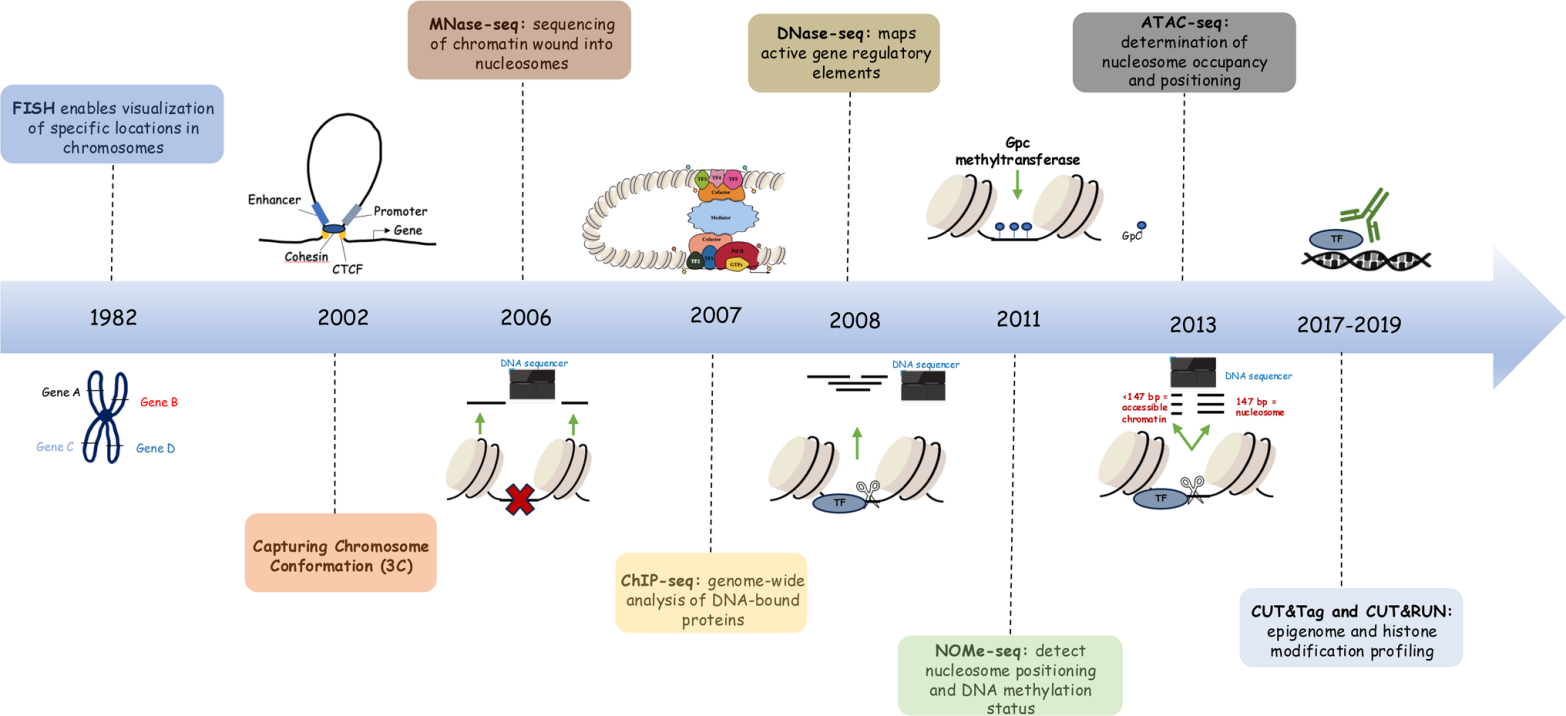
A timeline of the evolution of chromatin analysis techniques. Discovered in 1982, fluorescence in situ hybridization (FISH) allows researchers to detect and map specific DNA sequences on chromosomes by using specially designed fluorescent probes. 20 years later, Chromatin Conformation Capture (3C) was developed, enabling the analysis of the overall spatial organization of chromosomes. Micrococcal nuclease sequencing (MNase-seq, 2006) utilizes micrococcal nuclease enabling the next generation sequencing (NGS) analysis of chromatin wound into nucleosomes. In 2007, a breakthrough discovery, chromatin immunoprecipitation followed by sequencing (ChIP-seq), allowed for genome-wide analysis of proteins bound to DNA, thus providing insights into epigenetic mechanisms. The development of DNase I hypersensitive site sequencing (DNase-seq, 2008) and nucleosome occupancy and methylome sequencing (NOMe-seq, 2011) was followed, enabling the precise identification of the location of promoters and enhancers as well as the detection of the nucleosome positioning and DNA methylation status, respectively. Transposase-accessible chromatin with sequencing (ATAC-seq, 2013) addressed many limitations, providing information about chromatin accessibility across the genome. Ultimately, Cleavage under targets and release using nuclease (CUT&RUN, 2017**)** and cleavage under targets and tagmentation **(**CUT&Tag, 2019) are two novel immunotethering methods for next-generation epigenomic profiling. (See also text in Section "The evolution of chromatin analysis techniques")

Until recently, our understanding of three-dimensional chromatin folding was limited due to restrictions in available techniques. However, given the fact that the chromatin landscape constantly changes, the development of a range of techniques to profile chromatin accessibility and functional interactions became essential (Fig. 2) [81, 82]. Such methods proved to be excellent tools for mapping regulatory elements across different cell types or states [83]. One of the most common cytogenetic techniques is fluorescence in situ hybridization (FISH), a method for the identification and visualization of specific chromosomal loci. Furthermore, chromosome conformation capture (3C) and its derivatives help investigate chromosomal structure [84]. Assay for transposase-accessible chromatin with sequencing (ATAC-seq), DNase I hypersensitive site sequencing (DNase-seq), micrococcal nuclease sequencing (MNase-seq) and nucleosome occupancy and methylome sequencing (NOMe-seq) are all methods used to study chromatin accessibility at a genome-wide level [81], while chromatin immunoprecipitation sequencing (ChIP-seq) reveals interactions between DNA and specific transcription factors [85].

### Fluorescence in situ hybridization (FISH)

Discovered in the 1980s, FISH is a powerful technique allowing researchers to detect and map specific DNA sequences on chromosomes (Fig. 2) [86, 87]. The main purpose of this technique is to detect chromosomal abnormalities [88]. FISH employs specially designed fluorescent probes that hybridize to targeted chromosomal regions in fixed cells allowing for the results to be analyzed under the fluorescence microscope [89]. Genetic disorders caused by gene fusions, abnormal number of chromosomes or the loss of a chromosomal region can all be detected through FISH [90]. In addition, FISH has been essential for gene localization, significantly contributing to the success of the Human Genome Project and advancing our understanding of developmental biology [86]. Although FISH assists in the direct identification of the 3D positions of specific genomic loci, it is unable to systematically uncover the complex folding patterns of chromatin [87].

### Chromatin conformation capture (3C) and variations

 3 C is distinguished by its high-throughput ability to analyze the overall spatial organization of chromosomes (Fig. 2) [91]. In more detail, 3C-based methods utilize crosslinking and ligation to capture the proximity of two genomic loci in three-dimensional space [92]. Genome-wide 3 C (Hi-C), circularized 3 C (4-C) and carbon copy 3 C (5-C) are all 3 C variations that have greatly contributed to a better understanding of the structure of the genome [93–95]. Among them, Hi-C, a powerful and the most extensively studied derivative, combines 3 C and next-generation sequencing (NGS) and entails crosslinking of chromatin with formaldehyde, followed by restriction enzyme digestion, re-ligation and subsequent massively parallel deep sequencing [95]. Re-ligation occurs only for DNA fragments that are physically associated in 3D space, thereby providing insights into genome-wide chromatin interactions [95]. Notable discoveries based on Hi-C include chromatin loops, TADs and A/B compartments, thus advancing our understanding of chromatin’s hierarchical folding [1, 2, 96].

### Chromatin immunoprecipitation sequencing (ChIP-seq) and variations

ChIP-seq is a highly powerful and valuable tool in epigenomic research [85, 97]. This technique enables genome-wide analysis of DNA-binding proteins, chemical modifications of histone proteins or nucleosome positioning, thereby providing profound insights into the topological organization of DNA-associated proteins and their influence on phenotypic outcomes (Fig. 2) [98]. ChIP-seq allows for the selective enrichment of DNA sequences bound by the target protein, followed by precipitation of the fragments by using specific antibodies (either for the target protein or the histone modification) and subsequent massive parallel high-throughput sequencing of the enriched fragments [99–102]. This indispensable method offers significantly improved data compared to ChIP followed by hybridization to microarray (ChIP-on-chip). Specifically, ChIP-seq offers important information about gene regulation and epigenetic mechanisms including post-translational modifications of chromatin and histone variants, while ChIP-on-chip is not efficient at discovering unknown protein binding sites [103, 104]. Despite its remarkable capabilities, ChIP-seq has certain limitations such as the need for a high amount of input material and the requirement for high-quality antibodies to ensure both specificity and accuracy [85]. In addition to ChIP-seq, similar methodologies have emerged to address the complex organization of chromatin. For instance, ChIP-loop, a combination of 3 C and ChIP, identifies the proteins involved in DNA loop organization [105]. Notably, chromatin immunoprecipitation with paired-end tag sequencing (ChIA-PET) reduces non-specific interaction noise, often observed in ChIP-seq, and provides insights into long-range chromatin interactions, thus transcription regulation [106]. Finally, chromatin immunoprecipitation-polymerase chain reaction (ChIP-PCR) offers the highest resolution and accuracy to characterize histone modifications within defined regions of the genome [107]. To gain a deeper insight into unique mechanisms occurring in certain subpopulations, two independent groups developed single-cell ChIP-seq (scChIP-seq) [108, 109]. scChIP-seq utilizes a microfluidics system combined with single-cell DNA barcoding technologies, acquiring single-cell chromatin data [108]. This method enables the identification of activating (H3K4me3) or repressive (H3K27me3) marks for transcription, among single cells, providing important information about cellular heterogeneity [109]. Such tools pave the way for the treatment of heterogeneous diseases including cancer at the patient level following precision medicine principles [109].

### Methods for assaying chromatin accessibility

Physical access to DNA is defined as the extent to which chromatinized DNA is available for binding by DNA-binding factors [83]. This accessibility exists on a dynamic spectrum ranging from “closed chromatin” to “permissive chromatin”, the latter initiating chromatin remodeling to ultimately lead to the “open chromatin” state, where chromatin becomes entirely accessible [81]. Chromatin accessibility is measured by quantifying its sensitivity to either enzymatic cleavage or methylation by using endonucleases, which digest dsDNA, and ligation-mediated PCR [110].

DNase-seq, first described by Boyle et al., provides extremely high resolution for identifying open chromatin by combining traditional DNase I footprint with NGS (Fig. 2) [111]. The DNase enzyme cleaves the unprotected DNA sequences (euchromatin), while chromatin packed into nucleosomes (heterochromatin) remains intact [111]. The isolated fragments are then used as a template for library construction and subsequent sequencing via NGS [111]. In brief, DNase-seq effectively maps active gene regulatory elements, providing precise representation of their locations across the genome, including promoters and enhancers [112]. Similarly, formaldehyde-assisted isolation of regulatory elements (FAIRE-seq) provides insights into the regulatory activity of DNA regions in the genome [113]. In FAIRE-seq, DNA–protein complexes are crosslinked using formaldehyde [114]. Ultimately, sequencing identifies regions of low nucleosome density which are typically associated with genome regulatory areas, such as protein binding sites and promoters [113].

MNase-seq, first pioneered in the mid-2000s, utilizes micrococcal nuclease to cleave and eliminate the naked DNA, leaving chromatin wound into nucleosomes for NGS analysis (Fig. 2) [115, 116]. In fact, MNase-seq relies on the activity of the MNase enzyme which functions as an endo- and exo-nuclease and cleaves protein-unbound DNA [117]. Thus, DNA bound to histones or transcription factors remains intact [81]. Intact DNA is purified and NGS is performed to obtain the genomic sequences directly relevant to the epigenome [117, 118].

Chromatin accessibility and its epigenetic status can alternatively be assessed using NOMe-seq [119]. Specifically, NOMe-seq is based on the treatment of chromatin with a GpC methyltransferase (M.CviPI methyltransferase) to detect nucleosome positioning and the DNA methylation status (Fig. 2) [120]. Although DNA methylation naturally occurs at CpG promoter sites in the mammalian genome, GpC sites (methylated by M.CviPI methyltransferase during NOMe-seq) are also abundant throughout the genome [81, 121, 122]. By methylating only unprotected by nucleosomes or transcription factors DNA regions, NOMe-seq creates a nucleosome footprint and at the same time allows analysis of the endogenous methylation patterns [119]. Although NOMe-seq provides a quantitative view of chromatin accessibility, it requires a large number of sequencing reads to achieve sufficient depth across the genome [81].

Both DNase-seq and MNase-seq require large numbers of cells as input (in the order of millions), which limits their applicability [83]. In contrast, ATAC-seq can be carried out with significantly fewer cells (around 50,000) and does not require prior knowledge of epigenetic marks to determine chromatin accessibility across the genome [123]. The ATAC-seq methodology relies on the hyperactive transposase Tn5 to cleave DNA and ligate short oligonucleotides (adaptors) at the beginning or the end of the DNA fragments which are chromatin regions characterized by increased accessibility (Fig. 2). Genome-wide mapping is followed by PCR amplification, and ultimately, the resulting double-stranded fragments are sequenced via high-throughput sequencing [124]. Notably, data from ATAC-seq experiments can be classified into two main categories: shorter reads (< 147 bp) which indicate nucleosome-free regions representing accessible chromatin, and longer reads (~ 147 bp) which reflect nucleosome regions, blocking the activity of the transposase [125]. Thus, ATAC-seq allows for the determination of both nucleosome occupancy and positioning [126]. Since Buenrostro et al. introduced ATAC-seq in 2013, it has gained wide popularity due to its advantages, such as simplicity and faster execution compared to other chromatin accessibility methods [127]. The method was further advanced in 2015, to study the chromatin accessibility profile at the single-cell level [128]. scATAC-seq is a droplet-based method utilized in individually barcoded cells from a sample and is characterized by high sensitivity [128]. This novel and cost-effective technique surpasses previous limitations and reveals the unique features of each cell that contribute to tissue function, development and various diseases [129]. However, scATAC-seq data are highly noisy and sparse compared to single-cell RNA-seq (scRNA-seq), thus requiring new approaches and software tools for their analysis [130].

### Novel immunotethering approaches for chromatin profiling

In recent years, two novel immunotethering methods using enzymatic fusion proteins have been introduced in the field of genomics and epigenomics (Fig. 2). Cleavage under targets and release using nuclease (CUT&RUN**)** and Cleavage under targets and tagmentation **(**CUT&Tag) address many limitations associated with traditional mapping methodologies such as ChIP-seq [131]**.** In more depth, CUT&RUN isolates DNA fragments associated with specific proteins and selectively cleaves antibody-bound chromatin [132, 133]. CUT&RUN has been used primarily to profile global transcription factor binding and histone modifications in mammalian cells [134, 135]. Similarly, CUT&Tag is another powerful assay for next-generation epigenomic profiling, overcoming the need for adapter ligation during library preparation, enabling single-cell and multiplexing applications [131].

## Chromatin organization in cellular senescence

### Overview of cellular senescence

**Fig. 3:**
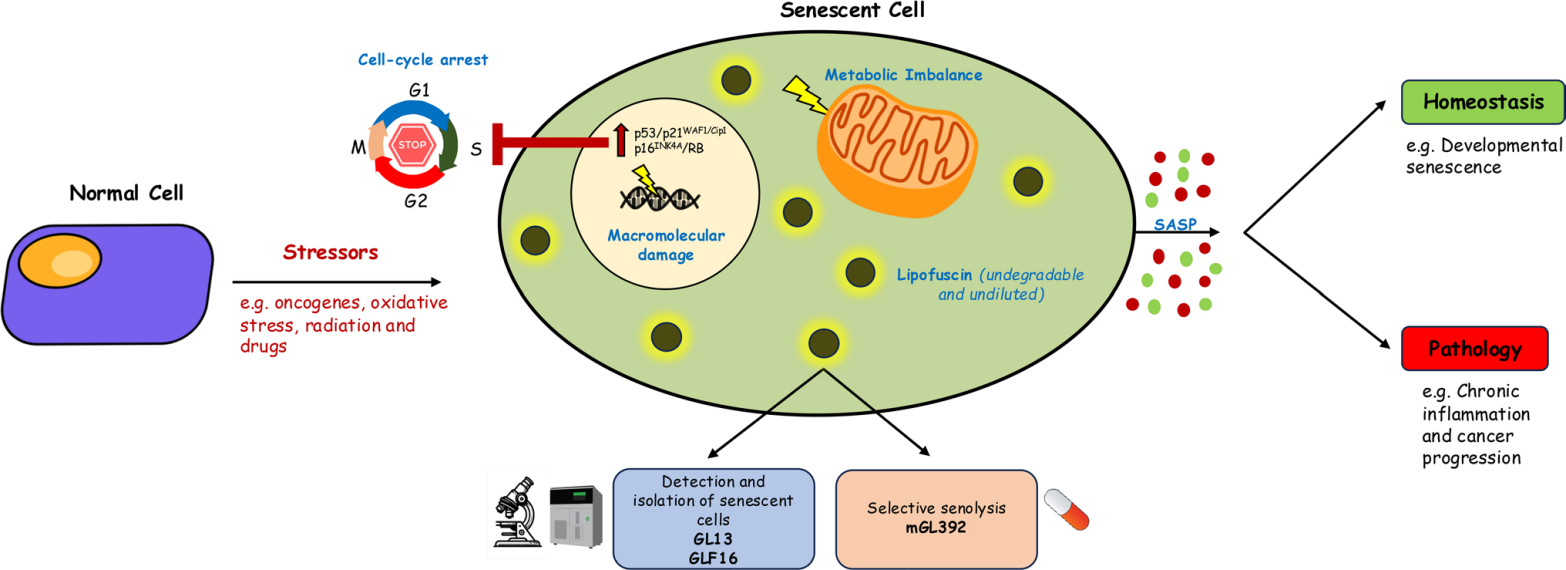
Hallmarks of senescent cells. Senescent cells exhibit four key hallmarks: cell cycle arrest, macromolecular damage, senescence-associated secretory phenotype (SASP) and deregulated metabolism. The accumulation of the heterogeneous fluorescent aggregate known as lipofuscin (the “dark matter” of senescent cells) has become a universal and reliable marker of cellular senescence. Therefore, two Sudan-Black-B (SBB) analogues have been developed, enabling the detection, isolation and tracking of live senescent cells (GL13, GLF16). mGL392 is a first-in-class senolytic platform that allows selective elimination of senescent cells by detecting lipofuscin. mitochondrium-1 icon by Servier https://smart.servier.com/ is licensed under CC-BY 3.0 Unported https://creativecommons.org/licenses/by/3.0/

Cellular senescence is a homeostatic mechanism activated in response to a variety of stressors [136]. On a temporal basis, it promotes the clearance of dysfunctional cells by the immune system during physiological processes such as embryonic development and wound healing, while its persistence is associated with chronic inflammation fueling the onset of various age-related pathologies including cancer, cardiovascular and neurodegenerative diseases (Fig. 3) [136–139]. Although senescence and aging are discrete entities, with the latter referring to the progressive decline in organismal functionality over time, senescence mirrors aging at the cellular level [136].

In 1961, Hayflick and Moorhead discovered that human fibroblasts lose their ability to proliferate after a finite number of cell divisions, providing the first evidence of senescence, termed replicative senescence (RS) [140]. Subsequent investigations clarified that this phenomenon relied on telomere shortening and dysfunction (damage) activating the DNA damage response (DDR) pathway and eventually resulting in cell cycle arrest [141]. Nowadays, it is well established that many stress insults such as oncogenes, DNA damaging agents and drugs, oxidative stress and metabolic perturbations can trigger senescence independently from telomere erosion, known as stress-induced premature senescence (SIPS) [142]. Although SIPS is not characterized by telomere attrition, it commonly features DNA damage at telomeric regions that fuel a persistent DNA damage response, implying thus also the involvement of telomere-related processes [142]. Oncogene-induced senescence (OIS), the most widely studied subtype of SIPS, is a well-known antitumor barrier, inhibiting the propagation of incipient cancer cells at the early stages of cancer development [143–146]. Oncogenes that trigger OIS include RAS, BRAF, CDC6, AKT, CYCLIN E and E2F1 [147, 148]. In addition, loss of tumor suppressors such as PTEN also induces cellular senescence as a failsafe mechanism against cancer, through the activation of the Akt pathway [147].

Senescence is an extremely dynamic and heterogeneous process [149, 150]. Early senescence pertains to a shift from a transient to a stable cell-cycle arrest where early senescent cells progress to ‘deep’ or ‘full’ senescence once SASP production is established [150]. Major differences have been identified between RS and SIPS at multiple levels such as mechanisms involved, gene regulation and time of occurrence during life [136]. For instance, one difference pertains to the differential accumulation of deregulated translation mechanisms such as ribosome stalling which appears absent in RS, in contrast to OIS [151]. Those differences may also be related to the epigenetic variability between senescence subtypes [152]. Despite the multi-faceted nature of the senescence phenotype, senescent cells exhibit four interconnected hallmarks: a) cell cycle withdrawal, b) altered metabolic function, c) macro-molecular damage, and d) a proinflammatory secretory property termed senescence associated secretory phenotype (SASP) [136, 153] (Fig. 3).

Senescence related cell cycle arrest is achieved through the sequential activation of the p53/p21^WAF1/Cip1^ and the p16^INK4A^/RB (retinoblastoma) axes [136]. p21^WAF1/Cip1^ expression is transient and is considered essential for establishing the senescent phenotype, while its maintenance is sustained through the persistent expression of p16^INK4A^ [154]. SASP, describes a unique secretome of pro-inflammatory cytokines (e.g. IL-1α, IL-1β, IL-6 and IL-8), growth factors (e.g. EGF and TGFα), chemokines (e.g. CXCL, CCL) and other inflammatory players (e.g. IFN-γ), that are cell-type dependent [155] and can act both in an autocrine and paracrine manner [156]. Early SASP facilitates normal processes eventually recruiting immune cells for senescence elimination following tissue regeneration, wound healing and embryonic development, while when its presence is indefinitely retained (late SASP) it becomes strongly pro-inflammatory, promoting disease development [149, 150]. An additional prominent feature of senescent cells is accumulation of macromolecular damage, which is directly linked to metabolic rewiring [136]. Apart from telomere shortening and subsequent DNA lesion accumulation [141], some of the most common types of damage are associated with protein [157] and lipid oxidation [153]. In parallel, key metabolic pathways such as glycolysis, autophagy, mitochondrial, and lysosomal metabolism are altered resulting in a deregulated metabolic profile [158]. Macromolecular damage contributes to increased reactive oxygen species (ROS) production, which exacerbates protein misfolding and aggregation, subsequently leading to metabolic disorders related to cellular senescence [159].

The most constant feature of senescent cells that reflects macromolecular damage, altered metabolic traits and cell cycle arrest is lipofuscin accumulation (Fig. 3) [136, 160]. Lipofuscin is a heterogeneous fluorescent aggregate consisting of oxidized proteins, lipids and metals [136]. Its specific aggregation during senescence has been exploited for the development of novel reagents that have been adopted in guideline multi-marker detection approaches, surpassing existing challenges in the field and uncovering the impact of senescence in human diseases and aging [136, 161–166]. Along with lipofuscin detection that is a prerequisite initial step, p16^INK4A^ and p21^WAF1/Cip1^ expression evaluation are subsequent steps of this algorithmic assessment [164, 165].

### Key players of the senescence epigenome program

**Fig. 4:**
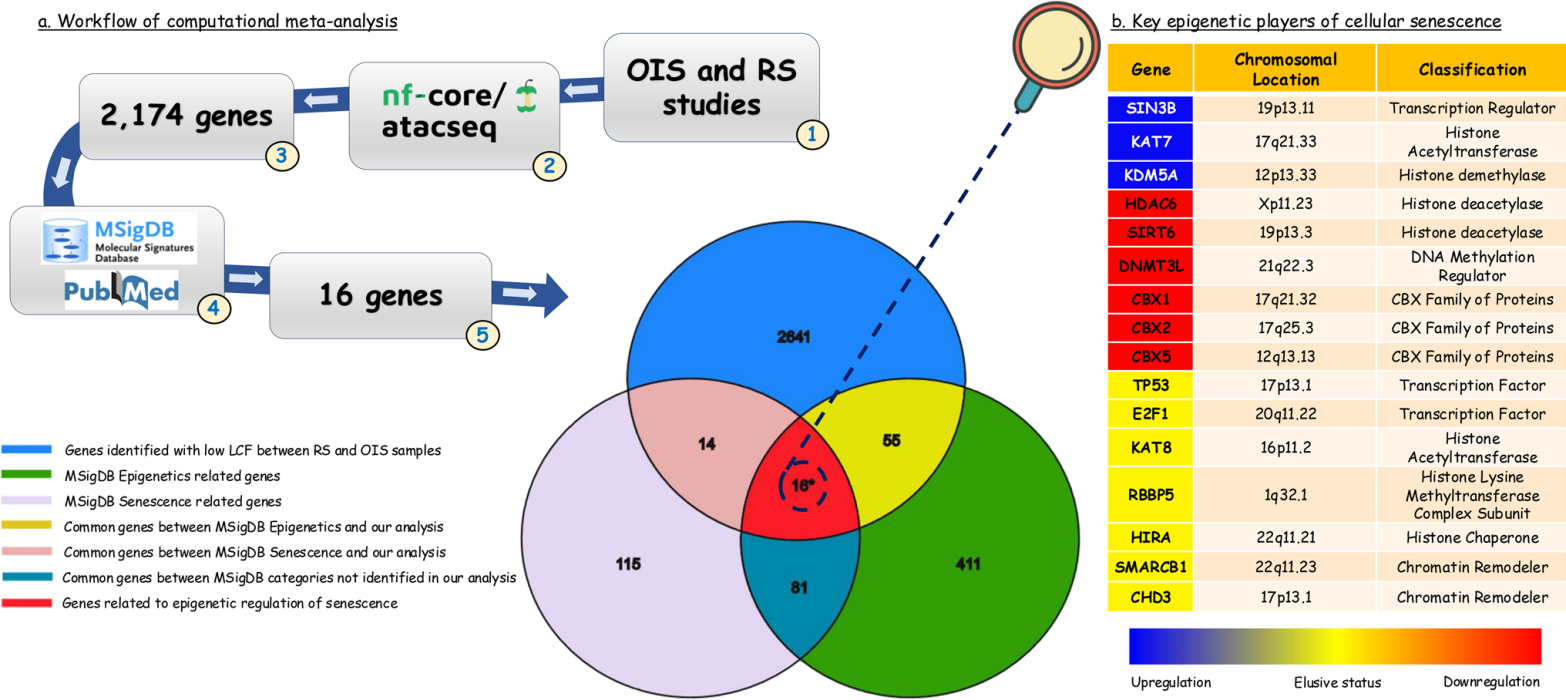
Computational meta-analysis in RS and OIS ATAC-seq data. The computational meta-analysis was conducted using the *nf-core/atacseq* pipeline and involved the comparison of three different studies of oncogene-induced senescence (OIS) and replicative senescence (RS). A total of 2.174 genes display unchanged expression between the senescence subtypes (log2 fold change lower than 1.5). By utilizing the Molecular Signatures Database (MSigDB) software and existing bibliographic data we identified 16 genes that are involved in the epigenetic regulation of senescent cells, as illustrated in the Venn diagram and accompanying Table in this figure. The 16 identified genes encode proteins that can be classified into the following categories: transcription factors/repressors (*TP53, E2F1, SIN3B*), histone modifiers (*KAT7, KAT8, HDAC6, SIRT6, KDM5A, RBBP5*), histone chaperones (*HIRA*), DNA methylation-associated proteins (*DNMT3L*), chromobox (CBX) family of proteins (*CBX1, CBX2, CBX5*) and chromatin remodelers (*CHD3, SMARCB1*). The locations of the 16 genes on their respective chromosomes and their association with cellular senescence are shown in the figure (See also text in Section"Key players of the senescence epigenome program")

In order to uncover key epigenetic regulators with a potential role in chromatin remodeling of cellular senescence, we conducted a computational meta-analysis by comparing three different studies of OIS and RS [167–169] (Fig. 4a). Raw ATAC-seq data from these studies were processed using the *nf-core/atac-seq* pipeline and analyzed to identify genes implicated in the epigenetic regulation of senescent cells. From a total of 2.758 genes identified to be expressed in senescent samples compared to controls, 2.174 display unchanged expression between the senescence subtypes. Moreover, using the Molecular Signatures Database (MSigDB) software, we identified 212 genes associated with the senescent program and 549 genes implicated in epigenetic regulation (Fig. 4a). Of these two distinct gene lists (senescence- and epigenetics-associated genes), we further identified a subset of 16 genes that are likely related to both processes, and are thus potentially involved in the epigenetic regulation of senescent cells (Fig. 4b). Indeed, according to the literature, several of these genes are inextricably linked to chromatin reorganization during cellular senescence, while others play an indirect role.

The 16 identified genes encode proteins that can be classified into the following categories: transcription factors/repressors (*TP53, E2F1, SIN3B*), histone modifiers (*KAT7, KAT8, HDAC6, SIRT6, KDM5A, RBBP5*), histone chaperones (*HIRA*), DNA methylation-associated proteins (*DNMT3L*), chromobox (CBX) family of proteins (*CBX1, CBX2, CBX5*) and chromatin remodelers (*CHD3, SMARCB1*). Among these genes, the expression levels of *SIRT6, DNMT3L, HDAC6*, *CBX1* and *CBX5* were found to be decreased while *SIN3B, KAT7* and *KDM5A* were overexpressed in senescence. Regarding the expression status of *TP53, E2F1, KAT8, RBBP5, CHD3, SMARCB1* and *HIRA,* it still remains elusive as they display context-dependent or dual roles in senescence (Fig. 4b).

One of the top hits of our analysis for epigenetically modulated genes in cellular senescence was the tumor suppressor p53. p53 plays a pivotal role in safeguarding genomic stability by functioning as a transcription factor that activates or represses multiple target genes [170]. Upon its activation, in response to DNA damage, p53 displays a dual role by promoting or inhibiting cellular senescence, depending on the cellular context, as well as the intensity and the persistence of the DNA damage [171]. On one hand, p53 transcriptionally regulates p21^WAF1/Cip1^ at the initial phases of cellular senescence [172]. On the other hand, p53 suppresses cytoplasmic chromatin fragments (CCF) that represent damaged nuclear chromatin marked by γ-H2A.X and are characteristic of senescent cells [173]. This contributes to restriction of the SASP phenotype via chromatin remodeling of SASP genetic loci [173, 174]. Ultimately, upon its activation, p53 rapidly induces 3D chromatin conformational changes, including changes in genome compartments, DNA loops and TADS [175]. Another transcription factor was E2F1 which is a critical modulator of cellular senescence as it regulates cell cycle progression [176]. During cellular senescence, the stable repression of E2F target genes is achieved through senescence-associated heterochromatin foci (SAHF) formation [177]. SAHFs are specialized domains of facultative heterochromatin, a type of heterochromatin capable of shifting from a condensed transcriptionally inactive to a loose active state depending on the cell’s needs [178]. SAHF formation coincides with the recruitment of heterochromatin proteins and of the retinoblastoma (Rb) tumor-suppressor to E2F-responsive promoters [177]. It was also shown that during cellular senescence, Rb recruits and cooperates with HDAC1 to repress E2F-regulated promoters of target genes involved in cell-cycle progression and mitosis [179]. SUV39H1, a histone methyltransferase, is also responsible for the repression of E2F activity in senescent cells [180]. In agreement with several studies, our analysis also highlights the role of the chromatin-associated SIN3 transcription regulator family member B (SIN3B) protein in both RS and OIS. Importantly, SIN3B overexpression induces senescence and promotes SAHF formation in Ras-induced senescence [181]. Specifically, upon oncogenic stress, SIN3B expression is found upregulated, leading to its recruitment to the promoters of E2F-target genes, which are consequently transcriptionally silenced [181].

KAT7 and KAT8 are key chromatin-modifying enzymes*,* both of which function as histone acetyltransferases. Interestingly, published evidence supports their capacity to modulate chromatin accessibility during cellular senescence. Genome-wide CRISPR-Cas9-based screens have identified KAT7 as a putative epigenetic driver of cellular senescence and its inactivation was linked to an extended lifespan in mice [182]. On the other hand, bulk RNA-seq data following the deletion of KAT8, an essential acetylating enzyme of histone H4K16 [183], uncovered the enrichment of pathways implicated in cellular senescence, such as p53 [183]. Additionally, the expression of another chromatin modifying enzyme, HDAC6, is significantly reduced in RS and its selective inhibition by N-acylhydrazone (NAH) induced senescence in carcinoma hepatocellular cells [184]. Sirtuin 6 (SIRT6), a nicotinamide adenine dinucleotide (NAD^+^)-dependent deacetylase was additionally identified in our bioinformatics analysis as an important epigenetic regulator of various cellular processes, including senescence [185]. Specifically, SIRT6 is capable of deacetylating specific sites of histones H3K9, H3K56 and H3K18, thereby inhibiting the activation of transcription factors implicated in senescence, ultimately preventing cells from entering senescence [185]. Consequently, senescent cells were found to exhibit decreased levels of SIRT6, leading to disruption of genomic integrity, telomere attrition and deregulated cellular homeostasis [185]. We further uncovered lysine demethylase 5 A (*KDM5A)*, a transcriptional repressor responsible for H3K4 demethylation at tumor suppressor genes, which promotes the growth of multiple human cancer types [186]. By blocking the KDM5A-H3K4me3 interaction, p16^INK4A^ activated G1 phase cell cycle arrest and cellular senescence [186]. Our meta-analysis also identified RBBP5, an H3K4 methyltransferase that is a core component of mixed-lineage leukemia 1 (MLL1) complex. Upon incorporation into this complex, RBBP5 mediates p16^INK4A^ activation and the establishment of the senescent phenotype in fibroblasts [187].

Importantly, we highlight the possible role of additional genes like *CBX1* (HP1β) and *CBX2,* in the epigenetic regulation of cellular senescence. Notably, senescent cells display lower levels of CBX1*,* while loss of CBX2*,* which is part of the polycomb repressive complex PRC1, leads to genomic instability as well as senescence-associated chromosomal rearrangements [188, 189]. *CBX5* encodes the non-histone chromatin-associated heterochromatin protein 1α (HP1α), which serves a key role in maintaining heterochromatin higher-order organization [190]. Initially, HP1α acts as a multivalent architectural protein which ensures genome integrity by promoting chromatin remodeling, leading to the activation of the DDR pathway [191]. Interestingly, HP1α is also a well-established marker of SAHF [192]. Although SAHF formation is not universal (it is observed primarily in OIS), HP1α has been identified as crucial for maintaining chromatin structure during RS as well [190].

Among the identified genes, the histone chaperone cell cycle regulator (HIRA) is known to be required for histone and chromatin control in senescent cells [193]. Specifically, previous work has shown that HIRA co-localizes with heterochromatin protein 1 (HP1) proteins into promyelocytic leukemia (PML) nuclear bodies, prior to their incorporation into SAHF [194]. PML bodies participate in the sequestration, modification, and degradation of various proteins and their detailed description is provided in Section"Higher-order chromatin structures"[195].

DNMT3L, which interacts with DNMT3A and DNMT3B, and enhances de novo DNA methylation was identified by our analysis as a key regulator [196]. Interestingly, mouse embryonic fibroblasts (MEFs) derived from DNMT3L-knockout mice exerted premature senescence, with the latter being accompanied by reduced H3K9me3 and H3K27me3 levels, indicating chromatin alterations and derepression of senescence-associated genes [197]. Ultimately, SMARCB1, a tumor suppressor protein and subunit of the SWI/SNF chromatin modifying complex, may serve as a critical regulator of cellular senescence through chromatin remodeling, as its loss seems to promote senescence in cancer cells [198].

### The chromatin landscape of cellular senescence

**Fig. 5:**
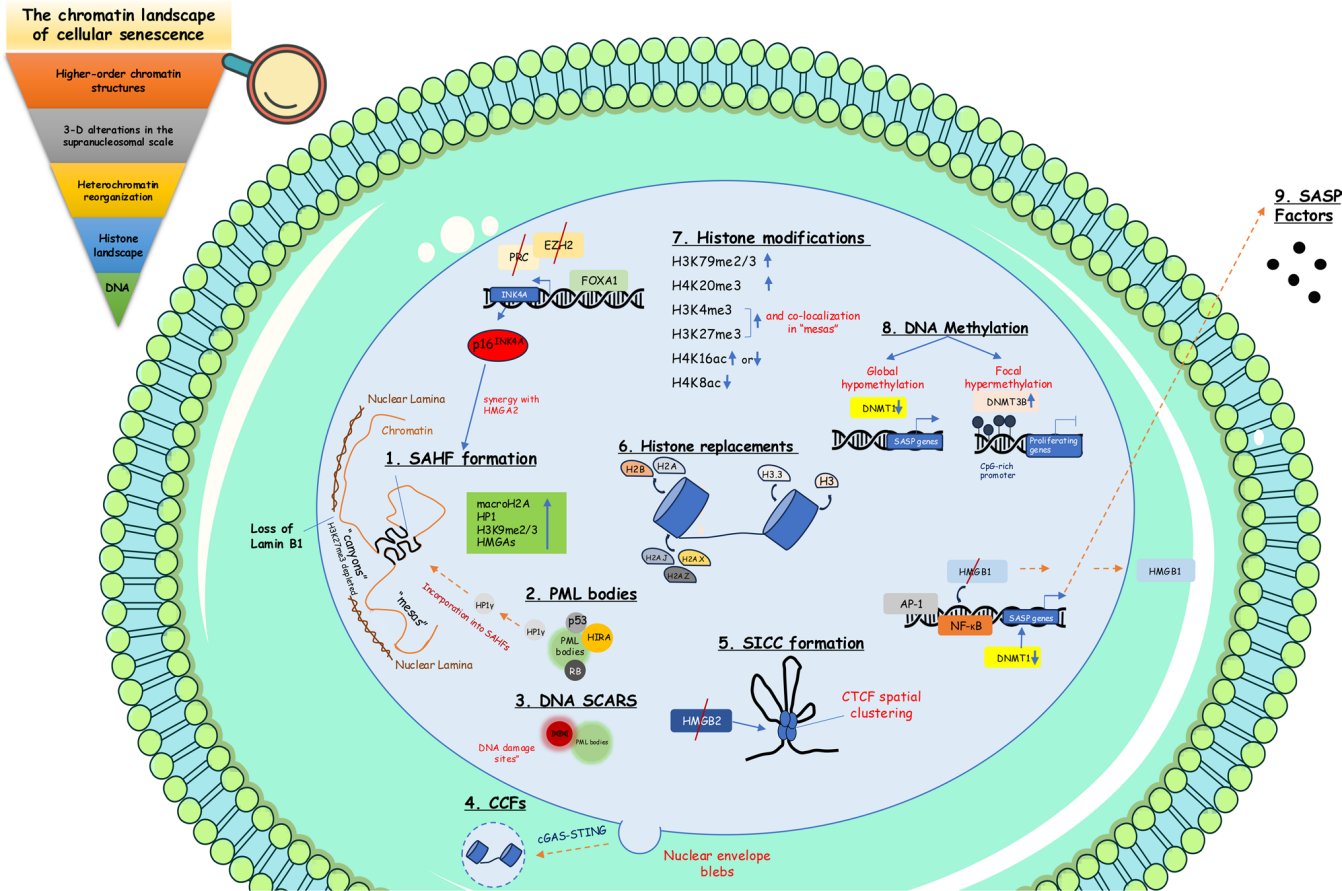
Key epigenetic mechanisms and large-scale chromatin events in senescent cells. Cellular senescence is characterized by distinct higher-order structures, including SAHFs (1), PML nuclear bodies (2), DNA-SCARS (3) and CCFs (4). At the supranucleosomal level, early senescence is marked by the loss of HMGB2, followed by senescence-induced CTCF spatial clustering (SICC) (5). In addition, p16^INK4A^ is regulated through a multi-level process, cooperates with HMGA2 protein and enhances the formation of SAHF, stabilizing cellular senescence. “Escape” from OIS is also regulated by chromatin loop reorganization (not shown in the figure). Moreover, the depletion of *LMNB1* leads to the rearrangement and relocation of heterochromatin from the nuclear boundary to its interior. Senescent cells exhibit reduction in core histones (H2A, H2B, H3 and H4) (6). Post-translational modifications of these histones such as H3K79me2/3, H4K20me3, H3K4me3, H3K27me3, H4K16ac and H4K8ac serve a pivotal role in the regulation of the senescent program and changes in their abundance are illustrated in this Fig. (7). Senescent cells are also associated with alterations in methylation status leading to changes in transcriptional regulation (8). Last, SASP is regulated by NF-κB and CCAAT/enhancer binding protein β (C/EBPβ) transcription factors, while the dissociation of sirtuin 1 (SIRT1) from the promoters of SASP genes enables their expression (9). “Image provided by Servier Medical Art (https://smart.servier.com/), licensed under CC BY 4.0(https://creativecommons.org/licenses/by/4.0/).”

In the following section, a comprehensive overview of the epigenetic program of senescent cells is provided, tracing chromatin architectural changes hierarchically. Starting from higher-order structures including SAHFs, PML nuclear bodies, “DNA segments with chromatin alterations reinforcing senescence” (DNA-SCARS) and CCFs, that are visible under the microscope, we conclude our analysis with lower-order elements like nucleosomes and their associated histones (Fig. 5).

#### Higher-order chromatin structures

A key feature of senescence is the presence of profound chromatin alterations, particularly at the level of chromatin interactions. Hi-C analyses have revealed conformational changes in chromatin organization in senescent cells compared to proliferating ones [199]. Specifically, in proliferating cells, heterochromatin is highly structured and deposited at the nuclear periphery, a feature that is lost in senescence [200] [201]. Moreover, it has been shown that aging is associated with large-scale spatial chromatin rearrangements [4]. A global shift in local interactions, particularly during SIPS, is marked by a decrease in interactions at repressive regions and an increase in long-range contacts with other heterochromatic regions [4]. This increase in distal interactions leads to both chromatin compaction and the formation of the so called senescence-associated heterochromatin foci (SAHF) (Fig. 5). Intriguingly, senescent cells are characterized by loss of lamin-dependent local interactions in SAHF heterochromatin that are accompanied by spatial clustering of constitutive heterochromatic regions, implying complex SAHF formation [201]. Comparison of embryonic stem cells (ESCs), somatic cells, and senescent cells shows a unidirectional loss in local chromatin connectivity, suggesting that senescence is an endpoint of a continuous nuclear remodeling process [201]. SAHF can be defined as chromatin-dense foci that contribute to the silencing of proliferating genes, mainly E2F-driven ones [178]. These compacted DAPI-stained foci are composed of various non-histone and histone chromatin proteins, such as macroH2A, HP1 and H3K9me2/3 [178]. However, SAHF formation and cellular senescence are not always coupled [178]. In fact, this varies considerably depending on both the senescence trigger and the cell type. For example, in c-Raf- and H-Ras- induced senescence, nearly all cells exhibit prominent SAHF, while CDC6, replicative and drug-induced senescence is not uniformly characterized by SAHF formation [178, 201, 202]. Yet, H-Ras did not form SAHF in breast MCF10A cells [203]. Furthermore, specialized domains of facultative heterochromatin are observed in extensively passaged primary human embryonic fibroblasts (IMR90 and WI38), while this is not the case in human foreskin fibroblasts (BJ cells), MEFs and HGPS cells of advanced passage numbers [178, 204]. Studies in mouse embryo fibroblasts and mouse skin fibroblasts showed increased levels of SAHF components, such as macroH2A, but the formation of SAHF per se is absent in these cell types [205]. Given that senescence and neurodegeneration are inherently linked, SAHF formation has also been observed in microglia or astrocytes [199]. Of particular interest are the high mobility group A (HMGA) and B (HMGB which will be discussed in Section"3-D alterations at the Supranucleosomal scale") family of non-histone chromatin proteins [206]. The role of HMGA proteins in both chromatin structure and gene regulation has been controversial. Initial studies emphasized the role of HMGA proteins in gene activation and proliferation, suggesting they could induce tumorigenesis [207]. However, it is now well-established that HMGA1 and HMGA2 have a dual role, as they are essential components of SAHF [208]. Various reports demonstrate deformation of SAHF upon inhibition of HMGA1 [209]. Within the same context, DNMT1-driven HMGA2 expression acts in synergy with p16^INK4A^ to promote SAHF generation and stabilization of the senescent phenotype (Fig. 5) [208]. HMGA proteins are also key drivers of SASP formation through various ways, such as chromatin remodeling, activation of signaling pathways and regulation of miRNA expression [206]. Specifically, HMGA drives the activation of the p38 MAPK/nuclear-factor-κB (NF-κB) signaling cascade leading to subsequent secretion of pro-inflammatory cytokines [210]. Moreover, HMGA proteins, through the activation of PI3K/Akt pathway, cause overexpression of matrix metalloproteinase 9 (MMP-9), a key factor of SASP and senescence-associated inflammation [211].

An additional higher-order chromatin change and prominent feature of senescent cells is the presence of PML nuclear bodies, structures where nascent RNA is synthesized and serve as a central hub of stress-induced cellular responses (Fig. 5) [212]. These dynamic nuclear structures, formed via liquid–liquid phase separation, are enriched in heterochromatin-associated proteins that repress E2F target gene expression, as well as proteins that are implicated in the induction of RS and OIS [213]. Furthermore, PML nuclear bodies facilitate DDR signaling and co-localize with the Rb and p53 tumor suppressors, thereby promoting a p53-dependent senescence pathway (Fig. 5) [213]. Ultimately, several studies suggest that PML bodies are involved in the formation of SAHF, as they recruit heterochromatin proteins such as HP1γ during early senescence, proteins that are later found as components of SAHF (Fig. 5) [213].

Persistent DNA damage, commonly evident in senescent cells, leads to the formation of distinct nuclear structures known as “DNA segments with chromatin alterations reinforcing senescence” (DNA-SCARS) (Fig. 5) [214]. These structures associate with PML nuclear bodies and lack evidence of single-stranded DNA and DNA synthesis [214]. Unlike transient damage foci, persistent DNA-SCARS are not characterized by active DNA repair, as DNA repair proteins such as RPA and RAD51 are absent [214]. Instead, DNA-SCARS accumulate the activated forms of the DDR mediators checkpoint kinase 2 (CHK2) and p53 in order to maintain both p53-dependent cell cycle arrest and SASP, essential characteristics of the senescence phenotype [214]. Of note, depletion of their core component, H2AX, reduced DDR-dependent senescence and IL-6 secretion [214].

In both RS and SIPS, a fundamental trait is nuclear envelope (NE) blebbing [215, 216]. Particularly, senescent cells display altered nuclear morphology, leading to nucleo-cytoplasmic chromatin blebbing [216]. The phenomenon of nuclear blebbing results from damaged chromatin and the presence of γΗ2ΑΧ serves as a marker, indicating involvement of the DDR pathway [217]. These nuclear membrane blebs contain chromatin fragments that eventually translocate into the cytoplasm, forming CCFs (Fig. 5). CCFs are enriched in heterochromatin markers including H3K9me3 and H3K27me3 and trigger an immune response through the activation of the cGAS-STING signaling cascade promoting SASP secretion [215, 216]. Unlike DNA-SCARS, 53BP1 is absent from CCFs and acts as a negative regulator of their formation. Lastly, nuclear blebbing, a hallmark of the HGPS cellular phenotype, is the result of progerin formation that in turn affects the nuclear lamina [215].

#### 3-D alterations at the Supranucleosomal scale

Cellular senescence is accompanied by excessive three-dimensional (3D) spatial alterations in the genome. These include transition from B-to-A compartments, affecting regions that are significantly enriched and regulate SASP factors [218]. In contrast, transitions from A-to-B compartments, mostly influencing cell cycle genes, are observed at a lower frequency [219]. In this process, the SMC complex component condensin plays a significant role, facilitating the expression of senescence related genes. These transitions are highly conserved across different types of senescence [215]. At a lower level, changes in the internal organization of TADs, which involve switching of sub-groups of TADs have been identified, resulting in altered interactions within the senescent cell epigenome compared to that of proliferating or quiescent cells [220]. Moreover, rewiring of chromatin loops has been reported in the context of RAS-induced senescence [221]. ChIP-seq and Hi-C data indicate that the extensive loop reorganization occurs mostly due to the redistribution of cohesin which leads to alterations in enhancer-promoter interactions, contributing to activation of genes such as those involved in SASP (*IL1B*) [221]. A characteristic paradigm of the above-mentioned 3D chromatin changes regarding *INK4A* locus regulation and subsequent p16^INK4A^ expression during senescence (Fig. 5) [4]. In proliferating cells, the *INK4A* locus is suppressed by polycomb repressive complexes (PRC1 and PRC2), recruited through the ANRIL long non-coding RNA [222]. The locus is maintained in a silent state by the activity of BMI1 protein, a component of the Polycomb Group (PcG) proteins [223]. As a result, EZH2 histone methyltransferase, an element of the PRC2 complex, imposes the repressive H3K27me3 histone mark, forming a specific type of TAD called polycomb domain that is not conducive to enhancer-promoter interactions [224]. At the same time, chromatin looping formed by CTCF further reinforces repression of the *INK4A* locus, thus revealing the complexity of its chromatin-based control mechanisms [225]. In contrast, in senescent cells the opposite events including PRC delocalization, EZH2 transcriptional downregulation and subsequent loss of the H3K27me3 histone mark occur [4]. Additionally, downregulation of CTCF and subsequent disruption of chromatin loops, along with the binding of FOXA1 transcription factor to the *INK4A* locus, decreases nucleosome distribution and activates the expression of p16^INK4A^ [225].

Apart from senescence induction, chromatin loop reorganization also plays an essential role in the reverse phenomenon termed “escape” from senescence. We and others have demonstrated that senescent cells are able under circumstances to re-enter the cell cycle, challenging thus the long-held dogma of irreversible cell-cycle arrest that traditionally existed in the senescence field [226–229]. Characteristically, in a CDC6 driven OIS model, a 4-Mbp long chromosomal inversion on *chr3*, harboring the circadian gene *BHLHE40* was sufficient to drive escape from senescence. New loop emergence in this 4-Mbp region resulted in insulation of two existing central TADs (one of which harbors *BHLHE40*) from each other, driving the “escape” transcriptional program and reactivating proliferating genes [229–231]. “Escaped” cells were found to acquire the motile and invasive characteristics of mesenchymal cells, in line with the adoption of an epithelial-to-mesenchymal (EMT) transition program [226–230, 232, 233]. Wnt/β-catenin signaling is a key driver of EMT but also a fundamental feature of “senescence associated stemness” that has been shown to promote “escape” from therapy induced senescence (TIS) [226, 234]. All the above, along with the fact that *BHLHE40* can activate the Wnt/β-catenin pathway via long non-coding RNA NEAT1, implies that *BHLHE40* is a cardinal component of the “escape” program [235].

HMGB proteins, abundant in the nucleus, play crucial roles in DNA looping, unwinding and bending [236]. By binding at TAD boundaries, loops are formed modulating the spatial genome arrangement and gene expression [237]. As such, nuclear HMGB1 loss, linked with senescence, is accompanied by topological changes that favor the senescence transcriptional program [237]. Moreover, HMGB1 nuclear deprivation results from translocation to the cytoplasm and subsequent secretion, leading to NF-κB activation through Toll-like receptor signaling, eventually triggering SASP and paracrine senescence. Interestingly, senescence associated gene expression is also enhanced by the fact that HMGB1 functions as an indisputable RNA-binding protein that interplays with a variety of mRNAs, thus affecting the availability of senescence-related mRNAs [233]. For instance, by interacting with senescence-relevant mRNAs or even directly with pro-inflammatory cytokines (IL-1β and TNF-α), HMGB1 may enhance the levels of key SASP factors, including IL-6 and MMP-3 [237, 238]. Of note, similar to HMGB1, HMGB2 loss occurs early during senescence acquisition and is followed by senescence-induced CTCF spatial clustering (SICC) (Fig. 5) [239]. The latter results in the emergence of new, long-range CTCF-anchored chromatin loops which are often associated with activation of genes [219]. Paradoxically, upon HMGB2 depletion during OIS, SASP gene loci have been reported to be incorporated into SAHFs and become silenced, highlighting its complex and context-dependent role in senescence [240]. Despite the fact that HMGB2 levels decrease in senescent cells, a significant proportion of HMGB2 bound to chromatin has been reported, requiring further investigation on its role during senescence [239]. Many up-regulated SASP genes, including *IL-8* and *IL-6*, preferentially exerted increased HMGB2 association that was not mediated by NF-κB [240]. Furthermore, HMGB2 drives H3K4 trimethylation, a process mediated by the methyltransferase MLL1, which is a critical epigenetic activator of SASP and DDR [241].

#### Heterochromatin reorganization

In normal proliferating cells, heterochromatin is anchored to the periphery of the mammalian nuclei at the nuclear lamina via lamina-associated domains (LADs), which harbor mostly repressed genes and heterochromatic histone marks [242]. The nuclear lamina is composed of A- and B-type lamins, along with other associated proteins [243]. Cell cycle arrest and macromolecular damage, hallmarks of senescent cells, are likely primary causes of lamin B1 (*LMNB1)* deletion, though further investigation is required to fully elucidate that link [244]. Since lamin B1 loss typifies all types of senescence, it is considered a notable senescence-associated biomarker [245]. Its depletion has dramatic consequences, leading to the rearrangement and relocation of heterochromatin from the nuclear boundary to its interior [246]. This affects lamina structure, chromatin organization and nuclear morphology [245]. The reorganization induced by lamin-B1 loss is responsible for the formation of “mesas” and “canyons”, as discussed below [243]. Interestingly, *LMNB1* restoration in already replicative-induced senescent cells does not result in the re-entry of the cell cycle or the re-establishment of LAD deposition suggesting that irreversible events have already occurred [247]. While the majority of LADs are conserved across cell types and differentiation states, these dynamic features are restructured in senescence, inducing rewiring of both short and distal chromosomal interactions and their association with nuclear lamina [242]. Specifically, the loss of *LMNB1* results in breakdown of the nuclear lamina and the subsequent repositioning of LADs away from it, mostly in regions that are rich in AT content and H3K9me2/me3 (Fig. 5) [248]. The translocation of LADs to the interior of the nuclear space is heavily associated with the formation of SAHF [219].

The packaging of the genome to form heterochromatin carrying H3K9me3 and H4K20me3 marks, ensures both gene silencing and genome stability in proliferating cells [249]. Heterochromatin is highly enriched in repetitive sequences such as centromeric and telomeric repeats, as well as transposons [250]. However, in cellular senescence, a global erosion of constitutive heterochromatin is observed, accompanied by instability at telomeres and centromeres, as well as derepression of retrotransposons [251]. Late passage human fibroblasts exhibit both telomere attrition and relocalization to the nuclear center, unlike OIS where telomeres are mostly associated with the nuclear lamina [252, 253]. Centromeres also exhibit dramatic structural changes during cellular senescence. Centromere protein A (CENP-A), a histone variant, plays a crucial role in protecting centromere integrity, and senescent human fibroblasts display reduced CENP-A levels [215, 254]. Additionally, heterochromatin loss is indicated by decreased levels of H3K9me3 in senescent fibroblasts derived from progeroid mice and in human mesenchymal stem cells (MSCs) from HGPS [255]. Interestingly, loss of H3K9me3 histone mark has been observed in the excitatory neurons of aged mice through single-cell epigenomic profiling methods (including scATAC-seq), revealing cell-type-specific-changes [256]. H3K9me3 depletion is also reported in Cockayne syndrome, a rare autosomal recessive neurodegenerative/progeroid disorder, due to downregulation of SUV39H1 and SETDB1 methyltransferases. A similar pattern is observed in fibroblasts following bleomycin treatment [257, 258]. Likewise, lamin A (*LMNA)* deficiency and the depletion of the DNA repair gene *WRN,* which occurs in Werner Syndrome (WS) and causes premature aging, leads to H3, SETDB1, SUV39H1 and HP1 deregulation, contributing to heterochromatin loss and DNA damage [255]. Knocking down one of the most established markers of heterochromatin, HP1α, results in the induction of premature senescence, confirming that loss of heterochromatin contributes to cellular aging [215]. It is well known that prior to their relocation to SAHF, HP1 proteins co-localize with HIRA at PML nuclear bodies, and although HIRA’s role into PML bodies still remains unclear, inhibition of its entry blocks the formation of SAHF [194]. In RS, global epigenetic alterations include transcription and ultimately the transposition of major retrotransposon classes, such as Alu (associated with persistent DNA damage foci), SVA (consisting of SINE, Variable tandem repeats and Alu) and LINE-1, as chromatin in these regions becomes relatively more accessible [259]. In line with this notion, the transcriptional derepression of the retrotransposable element LINE-1 (L1) during senescence, leads to activation of type-I interferon (IFN-I) response [260]. The transposition of retrotransposons is facilitated by the loss of repressive marks and the activation of the transcription factor FOXA1, which may contribute to age-related pathologies, including cancer [259].

#### The histone landscape

The expression of core histones (H2A, H2B, H3 and H4) is replication-dependent, whereas most histone variants are assembled in a replication-independent manner [261, 262]. Senescence, replicative and stress-induced, is accompanied by considerable changes in histone composition [219, 263, 264]. Telomere shortening, the main mark of RS, appears to reduce the levels of H3 and H4 (Fig. 5) [265]. In addition, loss of the histone linker H1 and reduced expression of the histone chaperones ASF1A/B and CAF1-p150/p60 complements the core histone landscape alterations that portray senescence [266, 267]. The histone variant H3.3 accumulates during both RS and OIS, replacing H3.1 and H3.2. H3.3 is implicated in the downregulation of proliferative genes playing a crucial role in cell cycle arrest and establishment of the senescent phenotype [58, 268]. This process is regulated by the proteolysis of H3.3, with the resulting cleaved product, H3.3cs1, promoting transcriptional silencing of cell cycle regulators such as RB/E2F target genes [269]. The main histone chaperon responsible for the dynamic incorporation of H3.3 into DNA at specific genomic loci is HIRA, orchestrating the transcriptional activation of genes required for the establishment and maintenance of the senescence program [270]. As a result of continuous DNA damage, senescent cells accumulate the phosphorylated form of H2A histone family member X (γ-H2A.X) [271, 272] and the poorly characterized H2A variant, H2A.J [273]. H2A.J promotes SASP, indicating its potential as a biomarker for senescence [274]. macroH2A, an H2A histone variant, is a key molecular component of SAHF which mainly characterizes OIS [275]. Lastly, the H2A.Z variant, which in proliferating cells is differentially localized within the p21^WAF1/Cip1^ promoter and functions as a negative regulator, is evicted from the promoter in response to DNA damage, thereby enabling p21^WAF1/Cip1^ transcription and the establishment of cellular senescence (Fig. 5) [276].

Alterations in histone modifications play a crucial role in the regulation of the senescence program. Senescent human fibroblasts induced by Ras exhibit distinct nuclear rearrangements of both repressive histone marks H3K9me3 and H3K27me3, into structured layers [277]. This reorganization is pivotal for SAHF formation; however, the global levels of these modifications remain intact [277]. Furthermore, in OIS, a global rise in transcription-activating histone marks H3K79me2/3 is observed, attributed to the overexpression of the DOT1 like histone lysine methyltransferase (DOT1L) [278]. In all forms of senescence, the rate of repressive H4K20me3 is increased at SAHFs, due to the high activity of Suv420h2 [279, 280]. Notably, it has been shown that H4K20me3 levels increase with age [281].

In both RS and OIS, chromatin regions enriched in H3K4me3 and H3K27me3 form “mesas”, large regions of “bivalent” chromatin, which are associated with active and repressive chromatin modifications, implying a complex interplay of chromatin structures as the senescence program is implemented (Fig. 5) [243]. On the other hand, “canyons” are more accessible chromatin regions, deprived of H3K27me3 (Fig. 5) [243]. Loss of H3K27me3 in “canyons” is linked to upregulation of genes related to SASP [282]. “Mesas” are primarily located at lamin B1-associated domains, while “canyons” are located between LADs and are enriched in genes and enhancers [137]. The formation of “mesas” and “canyons” is a result of *LMNB1* depletion in senescent cells [243]. In line, as stated above, downregulation of the methyltransferase EZH2, observed during cellular senescence, results in a reduction of H3K27me3, which in turn activates the cyclin-dependent kinase inhibitor and potent senescence inducer p16^INK4A^ [283]. HGPS, characterized by features of an accelerated aging process, results from a single mutation in the *LMNA* gene [243]. In HGPS cells, premature formation of H3K4me3 “mesas” contributes to the senescence of these cells, thus driving the progression of the syndrome [243].

Emerging data suggest that epigenetic modifications at enhancers more robustly determine the program of cellular senescence compared to promoters, which are minimally affected [218]. ChIP-seq analyses have revealed that senescence-associated super-enhancers are enriched with multiple histone acetyl marks, including H3K27ac, H3K18ac, H3K122ac, and H4K5ac (Fig. 5) [284]. These acetylation marks are induced by HAT p300, a primary driver of RS [218, 281]. Additional studies on histone acetylation suggest that H4K16 exhibits increased levels of acetylation at specific genomic loci in both RS and SIPS, a result of reduced activity of the Sir2 deacetylase [285]. Lastly, a reduction in H3K9ac and H4K8ac upon exposure to hydrogen peroxide (H_2_O_2_) has been put forward as a fundamental characteristic of oxidative-induced senescence driven by transient alterations in the epigenetic regulation of histone deacetylation enzymes (Fig. 5) [279, 284, 286].

#### The DNA anatomy

DNA methylation underpins the presence of “epigenetic clocks” which involve a set of CpG sites whose methylation levels may yield an estimated epigenetic age [251]. Previous studies support the epigenetic drift model, suggesting that genomic hypomethylation plays a crucial role in aging [287, 288]. However, more recent studies using NGS or bisulfite sequencing indicate that global DNA methylation does not exhibit significant age-related changes [288]. These studies also do not report major changes in DNMT and ten-eleven translocation (TET) protein levels during aging and no evidence explicitly links DNA methylation pattern changes with extended lifespan [288]. With regard to senescence, some studies report that mainly RS, rather than OIS, is associated with epigenetic methylation-related cellular aging, primarily due to the decline in DNMT1 activity during RS (Fig. 5) [289, 290]. However, it remains unclear whether the hypomethylation observed in the enhancers of SASP genes is indeed important for their activation in paracrine senescence [218]. During RS, promoter hypermethylation of focal CpG islands, which may involve DNMTs other than DNMT1, suppresses genes involved in cellular biosynthesis and metabolism, contributing to a gradual decline of the biosynthetic processes (Fig. 5) [291, 292]. These epigenetic changes occur in near-senescent cells and limit their metabolic capacity, reinforcing thus the beneficial side of senescence as an anti-tumor barrier [148, 291].

#### Chromatin reorganization and SASP

As mentioned previously, the secretory phenotype of senescent cells is driven by the formation of new super enhancers, in contrast to the narrow typical enhancers found near the promoters of proliferating genes [282]. This rewiring activates the transcription of SASP genes, which are mainly controlled by the NF-κB, CCAAT/enhancer binding protein β (C/EBPβ) and activator protein 1 (AP-1) transcription factors (Fig. 5) [282]. This process is further enhanced by additional transcription factors, such as GATA4, which mediates the activation of NF-κB to initiate the SASP, facilitating senescence [293]. The transcription of SASP factors, IL-1β, IL-8 and IL-6 is enabled by the deposition of H3K9ac and H4K16ac at their promoters [218]. SASP genes are excluded from the heterochromatic regions of SAHF through a loop-based mechanism, allowing their accessibility and transcription [282]. Recent data indicate that in normal cells the deacetylase Sirtuin 1 (SIRT1) binds to the promoters of major SASP components, blocking their expression [294]. However, upon entering senescence, SIRT1 disassociates from these regions enabling their transcription [294]. Likewise, in OIS, the histone variant macroH2A1 acts either as a positive or a negative regulator of SASP gene expression [295]. Particularly, ataxia telangiectasia mutated (ATM)-dependent early macroH2A1 removal from chromatin regions encoding SASP genes enables their transcription, whereas ATM activation, triggered by ROS, forms a negative feedback loop, where macroH2A1 redistribution limits SASP expression to prevent excessive inflammatory signaling [295]. Another key transcription factor that drives the senescence transcriptional program via regulating the senescence enhancer landscape is AP-1. Indeed, AP-1 is essential for the expression of SASP genes in different cellular contexts (OIS and therapy-induced senescence or TIS) indicating that it is a master regulator of cellular senescence [169].

As the senescence program is imposed, significant epigenetic changes modulating SASP take place. Downregulation of lamin B1 facilitates the progression from early to late senescence states, through local and global modifications in chromatin methylation, enabling the expression of SASP genes [150]. Late phases are characterized by large scale alterations, promoting high SASP expression and heterogeneity [149, 150]. The transition to late senescence is achieved via histone replacement, chromatin budding and the activation of retrotransposons, thus effectuating the heterogeneity of SASP factors [150]. The heterogeneity of SASP is evident in various settings. It occurs at the inter- and intra- cell type level (Table 1). Examples of the former are seen in senescent MSCs, HSPCs, neurons and virus infected neural cells (Table 1) [149, 296–299]**.** Depending on the context, either a beneficial effect of the secretome is exerted or a detrimental one [149, 296–299]. For instance, with regard to the latter, perpetuating inflammation is seen in diabetic patients, harboring senescent HSPCs, where chromatin modifications (increased H3K4me1 and reduced H3K9me3) trigger transcription at the SASP loci [297]. Lastly, a paradigm of intra-cell type SASP heterogeneity was observed, by scATAC-seq, in aged non-cardiomyocytes [300].

**Table 1:** Inter- and intra-cell type heterogeneity in SASP expression among senescent cells

Cell type	SASP Factors/Levels	Outcome
MSCs[149]	Early senescence: IL-6 Late senescence: IFN, MMP3	Early senescence: Anti-tumor activity Late senescence: Anti-tumor or tumorigenic activity
Human HSPCs[296]	CCL3, CCL20, CSF3, IL1A, IL1B, IL1R1, IL6, TIMP1	Inflammation
HSPCs from diabetic patients[297]	IL6 and TNF	Diabetic cardiovascular and inflammatory complications
HSV-1 infected neural cells[298]	TNF, IL1β, TIMP1, MMP12, CCL2, CXCL2 and IL6	Neuroinflammation
Non-cardiomyocytes[300]	Macrophages: Highly heterogeneous SASP activity Fibroblast subcluster 6: CXCL1, IL6, TNF Endothelial cells: Low SASP activity and less heterogeneity Neutrophils: Heterogeneity over time	Cell-type specific cardiac aging

## Therapeutic interventions to regulate senescence

**Fig. 6:**
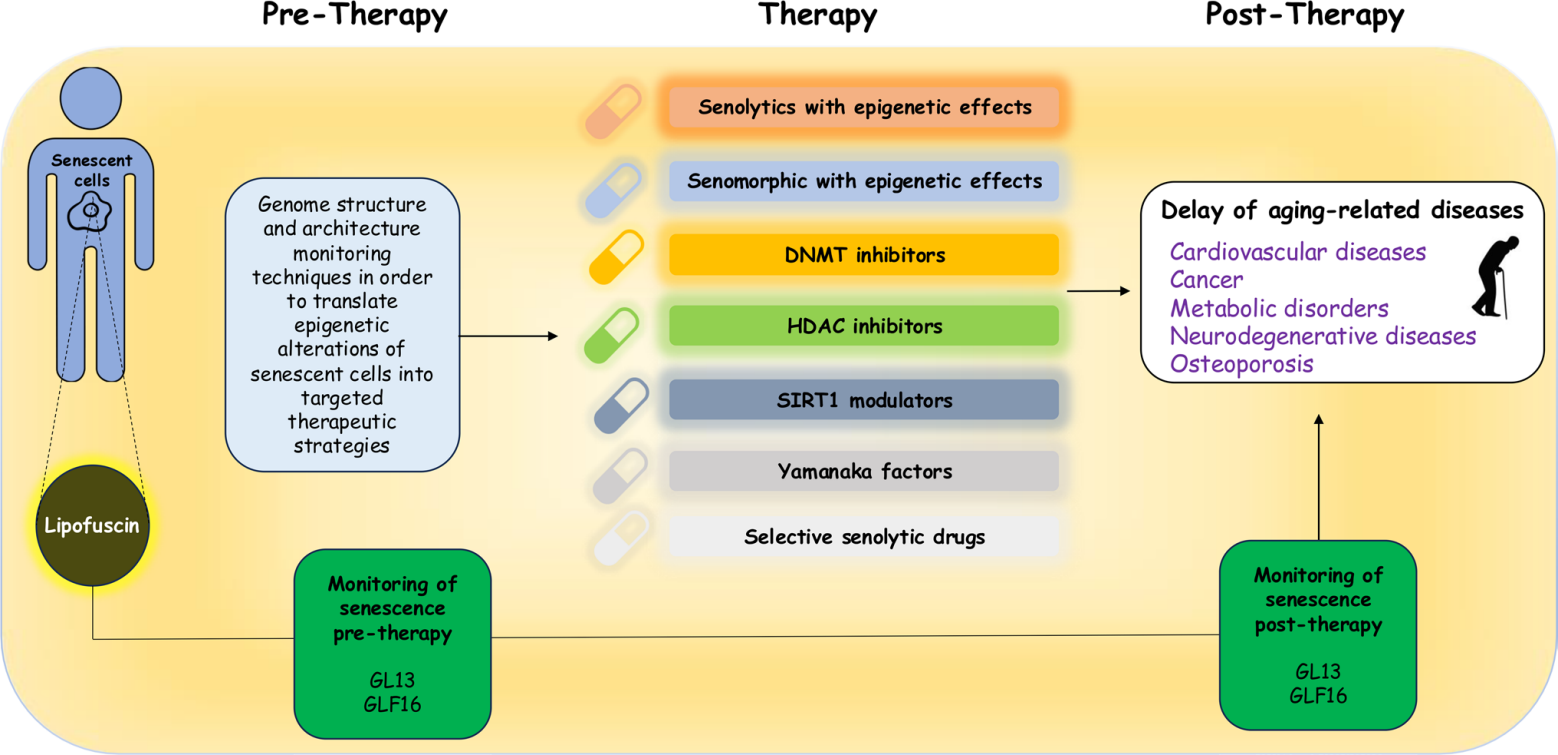
Epigenetic and senolytic therapies to delay age-related pathologies. Cellular senescence contributes to the development and progression of numerous diseases. Beyond senolytic and senomorphic drugs, there is increasing interest in leveraging the understanding of epigenetic alterations to develop targeted epigenetic interventions. These epigenetic modulators, summarized in Table 2, include senolytic and senomorphic compounds with epigenetic activity, SIRT1 modulators, DNMT and HDAC inhibitors as well as transcriptional reprogramming approaches involving Yamanaka factors. Likewise, by taking advantage of unique characteristics of senescent cells (lipofuscin), not only can selective senolytic drugs be designed, but also the monitoring of cellular senescence pre- and post-therapy can be achieved (GL13 and GLF16). Thus, delay of senescence-associated pathologies can be accomplished (See also text in Sections"Therapeutic Interventions to Regulate Senescence"and"Concluding remarks and future perspectives")

The ultimate goal in biomedicine is to capitalize on the developed knowledge for the generation of new effective therapeutic tools. In addition to senolytic drugs (which eliminate senescent cells) and senomorphic agents (which neutralize SASP), exploiting our awareness of the epigenetic changes may contribute towards targeted interventions that attenuate the onset or modulate senescence. However, many of these strategies are still in their infancy and accompanied by significant uncertainties. For the time being, the field relies on agents stemming from drug repurposing principles, which rather aim at non-specific mechanisms than at selective targets and exert significant side effects, highlighting the obstacles in the field [301]. Recently, the emergence of a first-in class senolytic platform (mGL392) paves the way to address these issues (Fig. 6) [302].

Despite the fact that the classification of epigenetic agents that exert anti-senescent effects presents significant challenges, Table 2 outlines promising classes of drugs exhibiting such potential. In brief, these reagents include senolytic and senomorphic drugs that act as epigenetic regulators, agents that inhibit DNMTs and HDACs, modulators of the anti-senescent factor SIRT1 and vitamin C (Fig. 6) [278, 303–316]. Likewise, therapeutic strategies targeting SAHF formation have attracted considerable attention [317, 318]. In addition to the aforementioned chemical-based strategies, rejuvenation of the transcriptional profile of senescent cells, by using the Yamanaka factors, is also gaining increasing interest [319, 320]. As also shown in Table 2, many of the above-mentioned epigenetic modulators have been tested in clinical trials with reported outcomes and side effects (https://clinicaltrials.gov/) (Fig. 6).

**Table 2:** Compounds that exert anti-senescence effects through epigenetic regulation

Compound	Category	Function or outcome at the epigenetic level	NCT Number	Adverse effects
Dasatinib & Quercetin	Senolytic	Reduce the expression of ZMAT2, chromatin rejuvenation [303]	NCT04063124 NCT05422885 NCT04313634	Low white blood cell count, pain
JQ1	Senolytic	Reduction of epigenetic age of leukocytes [304]	N/A (not available)	N/A
RG7112	Senolytic	Reduction of epigenetic age of leukocytes [304]	NCT00559533 NCT00623870 NCT01970930	No results posted yet
Nutlin-3a	Senolytic	Reduction of epigenetic age of leukocytes [304]	N/A	N/A
AMG232	Senolytic	Reduction of epigenetic age of leukocytes [304]	NCT03217266* NCT02110355 NCT01723020 NCT02016729	Syncope, anemia
BI01	Senolytic	Reduction of epigenetic age of skeletal muscle [305]	N/A	N/A
ABO	Anti-SAHF agent	Inhibition of ANXA7 and reduction of HP1γ [317]	N/A	N/A
ΒΗΒ	Anti-SAHF agent	Reduction of H3K9me3 levels [318]	NCT05732909	No results posted yet
RG108	DNMT inhibitor	Modulation of senescence-related genes through decreased DNA methylation [309]	N/A	N/A
SGI-1027	DNMT inhibitor	Antioxidant responses through the derepression of *KLOTHO* and subsequent activation of NRF2 [302]	N/A	N/A
Vorinostat	HDAC inhibitor	Inhibition of mTOR and NF-κB signaling pathways [311]	NCT03332667 NCT03212989 NCT02619253	Febrile neutropenia, procedural hypertension, anemia
Panobinostat	HDAC inhibitor	Chromatin relaxation, DDR activation and induction of apoptosis [312]	NCT02506959 NCT02717455 NCT02471430	Cardiac dysrhythmia, febrile neutropenia, infections, benign neoplasms
Fimenipostat (CUDC-907)	HDAC and PI3K inhibitor	Induction of apoptosis in the presence of p53 [313]	NCT01742988 NCT02307240 NCT02674750	Diffuse Large B Cell lymphoma, anemia, acute kidney injury
Resveratrol	SIRT1 modulator	Antioxidant responses through the activation of SIRT1 and subsequent activation of AMPK-FOXO3 [314]	NCT03253913 NCT03743636 NCT02523274	Infections, stroke, gastrointestinal issues, musculoskeletal disorders
Nicotinamide Riboside	SIRT1 modulator	Mitigation of oxidative damage [315]	NCT04818216 NCT04271735 NCT04078178	Death, respiratory failure
MLL1 shRNAs	Senomorphic	Reduction of *IL1β, IL1A, IL6, MMP1* and *MMP3* expression [306]	N/A	N/A
Anti-DOT1L agent	Senomorphic	Reduction of *IL1A* expression [278]	N/A	N/A
Anti-HMGB2 agent	Senomorphic	Reduction of SASP factors [307]	N/A	N/A
BRD4 inhibitor	Senomorphic	Reduction of SASP factors [308]	N/A	N/A
OCT4, SOX2, KLF4 and c-MYC (Yamanaka factors)	Transcriptional rejuvenators	Epigenetic reprogramming and de-differentiation [319, 320]	N/A	N/A
Vitamin C	Chromatin rejuvenator/Senomorphic	Rejuvenation of nuclear lamina and heterochromatin/Reduction of *IL-6* expression [316]	N/A	N/A

Gerotherapeutics, which focus on targeting the fundamental mechanisms of aging to prevent or delay age-related diseases and extend healthspan, is a pillar of geroscience. Epigenetic clocks that represent a practical tool in geroscience continuously emerge as promising biomarkers to measure biological age [321]. A number of clocks such as the Horvath’s clock, Hannum’s clock, DNA PhenoAge, and DNA GrimAge have been developed and provide insights into the prediction of mortality and various age-related pathologies by analyzing DNA methylation patterns at specific CpG sites [310, 322–324]. EpiTrace is a more sophisticated method which estimates the mitotic age of single cells using scATAC-seq data, thus providing not only insights into the biological age but further opening new horizons in the development of future clinical tools [325]. They have been importantly implemented in the evaluation of anti-aging interventions, which can reverse the epigenetic clock by cellular reprogramming or by pharmaceutical means [321]. In this context, of great interest are TET enzymes that serve a key role in the epigenetic regulation of several biological processes by oxidizing 5-methylcytosines (5mCs), thereby demethylating DNA [326]. Tet1 deficiency causes premature ovarian failure and leads to a reduction in spermatogonia stem cells and germ cell differentiation [327]. Thus, TET enzyme activation is being explored as a potential route to reset epigenetic clocks and delay senescence and biological aging [320]. Moreover, vitamin C, a direct regulator of TET activity, enhances the demethylation of DNA and promotes the expression of germline genes in mouse embryonic stem cells, further strengthening its role in the rejuvenation of the epigenetic landscape [328].

## Concluding remarks and future perspectives

Since the discovery of cellular senescence in 1961 by Hayflick and Moorhead, major advancements in understanding this phenomenon and its role in human diseases and aging have been achieved. Accumulating evidence suggests that due to ineffective removal by the immune system, senescent cells persist and progressively alter the tissue microenvironment, contributing to the onset of aging and age-related disorders [136, 165]. Interestingly, recent studies highlight the diverse role of senescent cells in different cancer types, where their progression depends on chromatin rearrangements during transcription or metabolic alterations [329, 330]. In essence, cellular senescence reflects aging at the cellular level [331]. Therefore, strategies dealing with removal of senescent cells (senotherapeutics) have emerged as attractive therapeutic opportunities. Toward this direction the senescent cell epigenome has gained increased attention. A plethora of sophisticated approaches and tools developed during the last decades have provided significant insights into key epigenetic events and large-scale chromatin remodeling processes within the senescence context, highlighting potential therapeutic targets and windows [218–220]. The most profound chromatin changes of senescent cells compared to proliferating ones, mainly comprise: i) nucleosomal alterations and ii) heterochromatin topology and chromatin interactions with the latter being characterized by a shift from local interactions to enhanced long-range contacts with other heterochromatic regions, leading to both chromatin compaction and the formation of SAHFs [4, 231]. SAHFs shuts-down the E2F-regulated cell proliferation network, contributing to cell cycle withdrawal, keeping in check the DDR pathway [332]. Nevertheless, although senescent cells are arrested, they exert constitutive DDR activation that continuously reshuffles their genome, driving chromatin reorganization. Eventually, these changes unlock at some point the replication machinery promoting cell-cycle re-entry with aggressive features [229]. This phenomenon termed “escape” acts as a source for tumor relapses and has raised the need of revisiting traditional anticancer therapies, to include approaches that eliminate senescent cells [230, 302]

However, this seems not the most favorable choice for all cell types and settings. For instance, senescent endothelial cells are difficult to be replaced upon their clearance by normal ones, in non-neoplastic settings, rendering the need for alternative therapeutic routes [333, 334]. These will aim to prevent the onset or neutralize senescence-related harmful outcomes. In this context, the SASP that is transcriptionally regulated by a variety of factors in an epigenetic manner, fueling the “dark” side of senescence, emerges as a promising target [218].

Considering all the above, epigenetic drugs, such as DNMT and HDAC inhibitors, and novel epigenetic modulators targeting key players of senescence, like those that came out from our analysis, could be exploited to alleviate the detrimental effects of senescence. For many years identification of senescence relied largely on the senescence-associated β-galactosidase (SA- β-Gal) assay [335]. The method though exerts significant drawbacks, rendering isolation of senescent cells for deep analysis not feasible [162, 164]. The finding that lipofuscin, the “dark” matter of the cell, is the only constant feature of senescent cells allowed the development of a series of reagents, rendering major challenges in the field addressable (Fig. 6) [136, 162, 164, 165, 302]. These developments will allow current and new directions to be explored more efficiently. Toward the latter, research in the fields of long non-coding and circular RNAs, as well as patient-derived organoids, may expand our therapeutic options at the personalized and epigenetic level [336–339].

## Materials and methods

### Bioinformatic meta-analysis

The *nf-core/atacseq* pipeline, a collection of standard industry tools, was utilized for each study with the default parameters, including the *narrow_peaks* flag, using as input the raw *fastq* files [340]. Downstream analysis of samples was performed in R programming language (version 4.4.2) using Seurat (version 5.2.1) and Signac (version 1.14.0) R packages [341–343]. Peak files from all 3 studies for each of the samples (52 samples) were combined into one list and processed using Seurat/Signac, removing zero-count peaks and calculating key metrics such as the strength of the nucleosome signal per cell (*NucleosomeSignal* function) as well as the TSS (*TSSEnrichment* function). Control samples were defined as samples with timepoints of 0 to 48 h from the OIS experiments as well as the *htert*-immortalized samples from the RS experiment, since they do not undergo replicative senescence. We estimated gene expression levels based on chromatin accessibility at promoter and gene body regions using *GeneActivity* function, while data were split based on the study of origin. Data were further processed by normalizing (*NormalizeData* function), identifying the top 2,000 Highly Variable Genes (*FindVariableFeatures* function), scaling (*ScaleData* function) and calculating Principal Component Analysis (*PCA*). Harmony integration strategy was used to integrate the data from the 3 different studies, while Uniform Manifold Approximation and Projection (UMAP) transformation was applied to the first 30 normalized Harmony PCA-derived components [344]. Samples, separated by study, were combined again using the *JoinLayers* function. Differentially expressed genes (DEG) were identified between control and senescent samples, as well as OIS versus RS senescent samples.

## Data Availability

All data reanalyzed in this study are available within the article. Any new data generated during the course of this study are available from the corresponding author upon reasonable request.

## Abbreviations

**3C**: Chromatin Conformation Capture
**4-C**: Circularized Chromatin Conformation
**5-C**: Chromosome Conformation Capture Carbon Copy
**5mCs**: 5-Methylcytosines
**AP-1**: Activator Protein 1
**ATM**: Ataxia Telangiectasia Mutated
**ATAC-seq**: Assay for Transposase-Accessible Chromatin with Sequencing
**BHB**: D-β-hydroxybutyrate
**C/EBPβ**: CCAAT/enhancer binding protein β
**CCF**: Cytoplasmic Chromatin Fragments
**CENP-A**: Centromere Protein A
**CHK2**: Checkpoint-2
**ChIP-PCR**: Chromatin Immunoprecipitation-Polymerase Chain Reaction
**ChIA-PET**: Chromatin Immunoprecipitation with Paired-End Tag sequencing
**ChIP-chip**: ChIP followed by Hybridization to Microarray
**ChIP-seq**: Chromatin Immunoprecipitation followed by Sequencing
**CpG**: 5′-C-phosphate-G-3′
**CTCF**: CCCTC-binding factor
**CUT&RUN**: Cleavage Under Targets and Release Using Nuclease
**CUT&Tag**: Cleavage Under Targets and Tagmentation
**DDR**: DNA Damage Response
**DEG**: Differentially Expressed Genes
**DNA-SCARS**: DNA Segments with Chromatin Alterations Reinforcing Senescence
**DNase-seq**: DNase I Hypersensitive Site Sequencing
**DNMTs**: DNA methyltransferases
**DOT1L**: DOT1 like Histone Lysine methyltransferase
**EMT**: Epithelial-to-Mesenchymal
**FAIRE-seq**: Formaldehyde-Assisted Isolation of Regulatory Elements
**FISH**: Fluorescence In Situ Hybridization
**HATs**: Histone Acetyltransferases
**HDACi**: HDAC Inhibitors
**HDACs**: Histone Deacetylases
**HGPS**: Hutchinson-Gilford Progeria Syndrome
**Hi-C**: Genome-wide 3C
**HIRA**: Histone Cell Cycle Regulator
**HMGA**: High Mobility Group A
**HMGB**: High Mobility Group B
**HP1**: Heterochromatin Protein 1
**HP1α**: Heterochromatin Protein 1α
**HPTMs**: Histone Post-Translational Modifications
**HSPCs**: Hematopoietic Stem Progenitor Cells
**K**: Lysine
**KDM5A**: Lysine Demethylase 5A
**LADs**: Lamina-Associated Domains
**LFC**: Log Fold Change
**LMNA**: Lamin A
**LMNB1**: Lamin B1
**MNase-seq**: Micrococcal Nuclease Sequencing
**MMP-9**: Matrix Metalloproteinase 9
**MSCs**: Mesenchymal Stem Cells
**MSigDB**: Molecular Signatures Database
**NCT**: National Clinical Trial
**NE**: Nuclear Envelope
**NF-κB**: Nuclear-factor-κB
**NGS**: Next Generation Sequencing
**NOMe-seq**: Nucleosome Occupancy and Methylome Sequencing
**non-CM**: non-Cardiomyocyte
**NR**: Nicotinamide Riboside
**NSCLC**: Non-Small-Cell-Lung Cancer
**OIS**: Oncogene-Induced Senescence
**PCA**: Principal Component Analysis
**PcG**: Polycomb Group
**PML**: Promyelocytic Leukemia
**PRC1**: Polycomb Repressive Complex 1
**PRC2**: Polycomb Repressive Complex 2
**RB**: Retinoblastoma
**ROS**: Reactive Oxygen Species
**RS**: Replicative Senescence
**RT**: Replication Timing
**S**: Serine
**SA-β-gal**: Senescence-associated β-galactosidase
**SAHF**: Senescence-associated Heterochromatin Foci
**SASP**: Senescence-associated Secretory Phenotype
**SBB**: Sudan-Black-B
**scATAC-seq**: Single-cell ATAC-seq
**scCHIP-seq**: Single-cell ChIP-seq
**SICC**: Senescence-Induced CTCF Spatial Clustering
**SIN3B**: Transcription Regulator Family Member B
**SIPS**: Stress-Induced Premature Senescence
**SIRT1**: Sirtuin 1
**SIRT6**: Sirtuin 6
**SMC**: Structural Maintenance of Chromosomes
**T**: Threonine
**TADs**: Topologically Associating Domains
**TET**: Ten-Eleven Translocation
**TIS**: Therapy-Induced Senescence
**TSS**: Transcriptional Start Sites
**UMAP**: Uniform Manifold Approximation and Projection
**WS**: Werner Syndrome
**Y**: Tyrosine
